# Single‐Cell RNA Sequencing Identifies MMP11^+^ Cancer‐Associated Fibroblasts as Drivers of Angiogenesis and Bladder Cancer Progression

**DOI:** 10.1002/advs.202502774

**Published:** 2025-06-24

**Authors:** Wuwu Xu, Ting Liang, Hu Fang, Lu Fu, Dashi Deng, Xiyang Tan, Lisha Liu, Dongdong Tang, Haoxiang Zheng, Qiuxia Ding, Xiuqi Hou, Daquan Feng, Tao Tao, Song Wu

**Affiliations:** ^1^ Urology Institute of Shenzhen University The Third Affiliated Hospital of Shenzhen University (Shenzhen Luohu People's Hospital) Shenzhen University Shenzhen 518116 China; ^2^ Shenzhen Following Precision Medical Research Institute Luohu Hospital Group Shenzhen 518000 China; ^3^ Shenzhen Key Laboratory of Digital Creative Technology the Guangdong Province Engineering Laboratory for Digital Creative Technology College of Electronics and Information Engineering Shenzhen University Shenzhen 518060 China; ^4^ The affiliated South China Hospital of Shenzhen University Shenzhen University Shenzhen 518000 China; ^5^ Department of Urology The First Affiliated Hospital of Zhengzhou University Zhengzhou 450052 China

**Keywords:** angiogenesis, bladder cancer, cancer‐associated fibroblasts, single‐cell RNA sequencing, tumor progression

## Abstract

Cancer‐associated fibroblasts (CAFs) play a crucial role in tumor progression, with heterogeneity influencing therapeutic response and prognosis, highlighting their potential as viable targets for treatment. In this study, a novel CAF subgroup, MMP11^+^ mCAF is identified, through single‐cell RNA sequencing, which accumulates progressively during bladder cancer progression and is significantly associated with poor prognosis. This cell population regulates the migration of tip endothelial cell clusters (ESM1^+^tEC) via the WNT5A‐MCAM signaling axis, and modulates the expression of key transcription factors, *SOX18*, *NFIC*, and *HOXB9*. Additionally, MMP11^+^ mCAFs recruit SPP1^+^ macrophages through CCL11/CCL2, promoting VEGFA secretion, which further enhances the pro‐angiogenic activity of ESM1^+^ tECs. Furthermore, interferon‐associated basal‐like tumor cells secrete BMP2, which induces the expression and activity of NFE2L3, a transcription factor specific to MMP11^+^ mCAFs, promoting *WNT5A* expression. Mouse experiments confirmed that inhibiting BMP2 can suppress tumor angiogenesis and growth in bladder cancer. Pan‐cancer analysis revealed that MMP11^+^ mCAFs are present across various cancer types, including breast cancer, lung adenocarcinoma, gastric cancer, and colorectal cancer. These findings provide insights into the heterogeneity of CAFs and their regulatory role in tumor progression, offering new potential therapeutic targets for CAF‐targeted treatments with broad applicability across cancers.

## Graphical abstract

Single‐cell RNA sequencing identifies MMP11⁺ CAFs as pro‐angiogenic cells accumulating during bladder cancer progression. These cells promote angiogenesis by regulating ESM1⁺ tip endothelial cells and recruiting SPP1⁺ macrophages. Tumor‐derived BMP2 promotes their activation. Targeting BMP2 suppresses tumor angiogenesis and growth, highlighting the therapeutic potential of CAF modulation in bladder cancer.

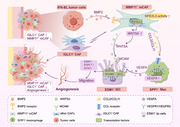

## 1 Introduction

Bladder cancer ranks among the ten most common malignancies worldwide, and its incidence is expected to continue rising globally. However, the 5‐year survival rate has shown little improvement over the years.^[^ ^1^, ^2^ ^]^ Conventional therapies for advanced cancer which primarily target cell proliferation or induce apoptosis, often fail to completely eradicate malignancies.^[^ ^3^ ^]^ Increasing evidence suggests that the tumor microenvironment (TME) plays a crucial role in tumor progression and therapeutic resistance—yet remain insufficiently addressed in current treatment paradigms.^[^ ^4^, ^5^ ^]^

Among the various TME components, tumor angiogenesis is particularly pivotal. It not only supplies oxygen and nutrients essential for tumor growth but also establishes vascular routes that facilitate tumor cell dissemination and metastasis.^[^ ^6^, ^7^ ^]^ Angiogenesis is a highly orchestrated process involving extensive crosstalk between endothelial cells, pericytes, vascular smooth muscle cells, tumor cells, tumor‐associated immune cells, and cancer‐associated fibroblasts (CAFs).^[^ ^8^ ^]^ Despite the clinical implementation of anti‐angiogenic therapies, their long‐term efficacy remains limited due to tumor adaptive mechanisms, activation of alternative pro‐angiogenic pathways, and the emergence of more invasive tumor phenotypes following treatment. Therefore, a deeper understanding of the regulatory mechanisms underlying tumor angiogenesis is essential for identifying novel therapeutic targets and improving treatment outcomes in bladder cancer.

CAFs are a major constituent of the TME and play a pivotal role in tumor progression through extracellular matrix (ECM) remodeling, cytokine secretion, and immune modulation.^[^ ^9^, ^10^, ^11^, ^12^, ^13^, ^14^ ^]^ Recent studies have highlighted their significant involvement in tumor angiogenesis. CAFs can directly promote neovascularization by secreting multiple pro‐angiogenic factors, including VEGF‐A, FGF2, and CXCL12.^[^ ^15^, ^16^, ^17^ ^]^ Additionally, CAFs indirectly facilitate tumor angiogenesis by recruiting and activating endothelial progenitor cells and myeloid‐derived cells within the TME, further accelerating tumor growth and invasion.^[^ ^18^ ^]^ Furthermore, CAF‐mediated fibrosis can alter the structural and functional properties of tumor vasculature, affecting vascular permeability and overall angiogenic dynamics.^[^ ^19^ ^]^ Notably, CAFs exhibit both pro‐tumorigenic and tumor‐suppressive functions depending on their specific subtypes and activation states. As a result, targeting distinct CAF subpopulations has emerged as a promising avenue for precision cancer therapy.^[^ ^10^, ^20^ ^]^ For example, selective targeting of PDGFRα^+^ ITGA11^+^ CAFs in bladder cancer has been shown to markedly reduce lymphovascular invasion and lymph node metastasis in early‐stage bladder cancer models.^[^ ^21^ ^]^ Likewise, inhibiting CCL2 signaling through ICAM1^+^ inflammatory CAFs effectively curtails tumor growth in murine bladder cancer models.^[^ ^22^ ^]^ These findings underscore the potential of therapeutic interventions aimed at specific CAF subtypes or their associated signaling pathways to advance bladder cancer treatment.

This study identified a novel fibroblast subset, MMP11^+^ mCAFs (matrix CAFs), which is associated with bladder cancer progression and poor prognosis. This subgroup resides in the perivascular region, promoting migration of ESM1^+^ tEC endothelial cells via the WNT5A‐MACM signaling pathway. Additionally, it recruits SPP1^+^ macrophages through the CCL2/CCL11 axis, promoting VEGFA secretion and regulating angiogenesis. This fibroblast population is regulated by BMP2 secreted by interferon‐associated basal‐like tumor cells. Mouse experiments confirmed that BMP2 promotes tumor progression, MMP11 expression, and angiogenesis. Pan‐cancer analysis further revealed that MMP11^+^ mCAFs are prevalent across multiple cancers, including breast cancer, lung adenocarcinoma, gastric cancer, and colorectal cancer, all correlated with poor prognosis. Our study emphasizes the role of the MMP11^+^ mCAF in bladder cancer and identifies a potential therapeutic target for its treatment. These findings advance our understanding of tumor heterogeneity in bladder cancer and provide a foundation for personalized therapy in urothelial carcinoma.

## 2 Results

### 2.1 A Geneset Associated with Advanced‐Stage Tumors and Poor Prognosis is Highly Expressed in Fibroblasts

**Figure 1:**
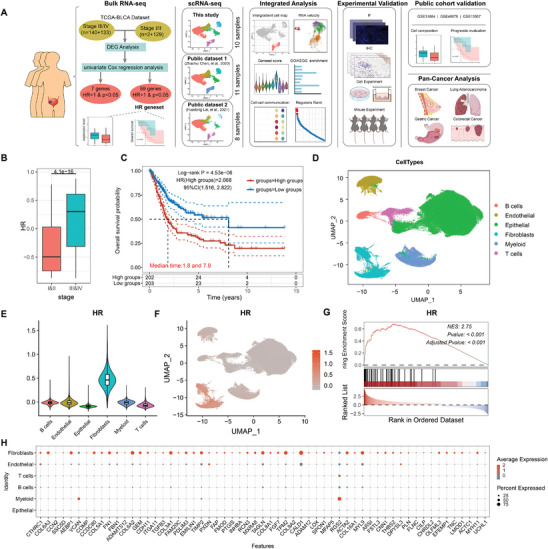
Identification of high‐risk (HR) gene set associated with bladder cancer progression. A) HR gene set identification and single cell analysis process. B) Expression of HR gene set in different stages of TCGA‐BLCA. C) Kaplan–Meier survival curves showing overall survival of HR gene set. Statistical analysis was performed using log‐rank test. High groups and low groups referred expression levels of HR gene set. D) UMAP layout of all cells belonging to the integrated dataset, colored by cell types. E,F) Violin plots (E) and UMAP (F) show the expression of HR gene set in different cell types. G) GSEA of HR gene set on genes ranked by log2 fold change between fibroblast versus other cells. H) Expression of genes in the HR gene set across different cell types.

Advanced‐stage tumors exhibit distinct transcriptional alterations compared to early‐stage tumors. To investigate this, we analyzed differentially expressed genes (DEGs) between stage III/IV and stage I/II tumors in the TCGA‐BLCA cohort, identifying 107 upregulated and 18 downregulated genes. Univariate Cox regression analysis further revealed that 59 upregulated DEGs were associated with poor prognosis, while 7 downregulated genes correlated with favorable outcomes (**Figure** **1**A; Figure S1A and Table S1, Supporting Information). These 59 upregulated genes were designated as the High‐Risk (HR) gene set, which showed significantly higher expression in stage III/IV tumors (Figure 1B). Kaplan‐Meier survival analysis confirmed that elevated HR expression was linked to reduced overall survival in the TCGA‐BLCA cohort (HR = 2.068, 95% CI = 1.516–2.822, *p* = 4.53e‐6) (Figure 1C).

To investigate the cellular expression pattern of the HR gene set, we constructed a single‐cell RNA sequencing library comprising 29 bladder tumors and adjacent normal tissues from our internal dataset, as well as two publicly available datasets (Table S2, Supporting Information).^[^ ^23^, ^24^ ^]^ After stringent quality control and batch correction, 184 996 cells were clustered into 29 subpopulations and classified into six major cell types based on established marker genes (Figure S1B–D, Supporting Information; Figure 1D). HR gene set signature score revealed that fibroblasts exhibited the highest expression among all cell types (Figure 1E,F), which was further supported by Gene Set Enrichment Analysis (GSEA) showing significant enrichment of the HR gene set in fibroblasts (Figure 1G). Examination of individual genes expression confirmed that most HR genes were predominantly expressed by fibroblasts (Figure 1H). These findings suggest that fibroblasts are the primary source of the HR gene set associated with poor prognosis in advanced‐stage bladder tumors.

### 2.2 The HR Geneset is Highly Expressed in MMP11^+^ mCAFs

**Figure 2:**
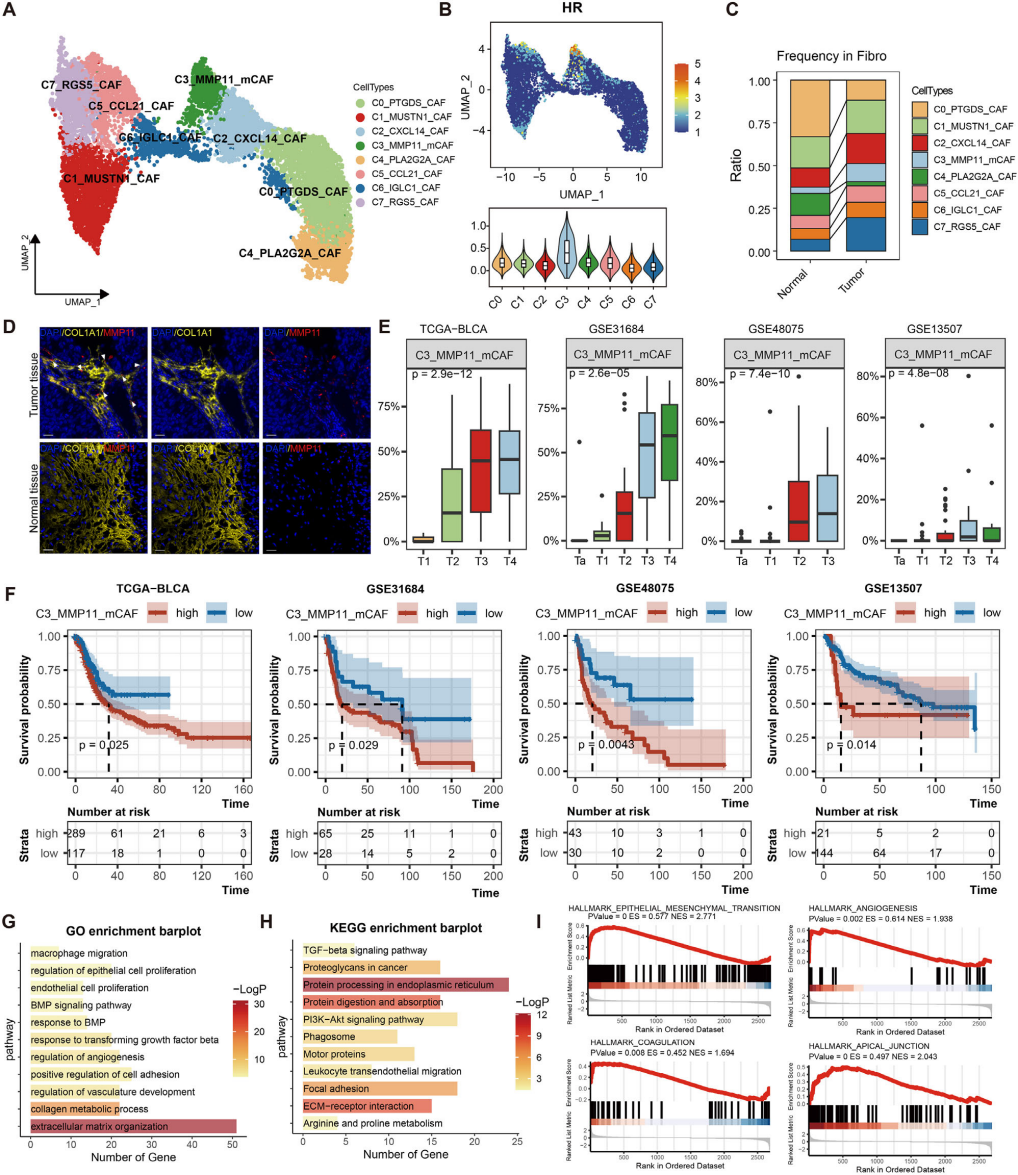
Identification of the fibroblast population MMP11⁺ mCAFs with high expression of the HR gene set. A) UMAP plot showing the classification of fibroblast populations. B) Expression of the HR gene set across all fibroblast populations. C) The proportion distribution of different fibroblast subpopulations in tumor and normal tissues. D) IF staining confirming the presence of MMP11^+^ mCAF cells in tumor tissues and normal tissues. Scale bar = 20 µm. The white arrow indicates the MMP11^+^ mCAF cells. E) The proportion of MMP11⁺ mCAFs in each clinical stage across four datasets. The box plots show the median (center line), the first and third quartiles (box boundaries), and the whiskers representing a maximum of 1.5× the interquartile range. Statistical analysis was performed using the Kruskal test. F) Kaplan–Meier survival curves illustrating the overall survival of patients stratified by the proportion of MMP11⁺ mCAFs across four bladder cancer cohorts. Statistical analysis was performed using log‐rank test. "High" and "Low" indicate groups with high and low proportions of MMP11⁺ mCAFs, respectively. G,H) Enriched GO (G) and KEGG (H) functions of upregulated genes in MMP11^+^mCAFs. I, GSEA shows top enriched pathways in MMP11^+^mCAFs.

To investigate the heterogeneity of fibroblasts, we isolated and reclustered fibroblasts population into eight distinct subclusters, each labeled based on its top marker genes (**Figure** **2**A; Figure S2A, Supporting Information). Among these, the MMP11^+^ mCAFs exhibited the highest HR signature scores (Figure 2B), with elevated expression of genes such as *COL1A1*, *MMP11*, *POSTN*, *COL3A1*, and *CTHRC1* (Figure S2B, Supporting Information). Proportional analysis revealed that MMP11^+^ mCAFs were more abundant in tumor tissues compared to adjacent normal tissues (Figure 2C). Immunofluorescence (IF) staining of tumor and adjacent normal tissue sections, using COL1A1 as a pan‐fibroblast marker and MMP11 as a specific marker,^[^ ^25^ ^]^ confirmed the predominant presence of MMP11^+^ mCAFs within the TME, supporting their potential functional relevance (Figure 2D).

To assess the clinical significance of MMP11^+^ mCAFs, we performed deconvolution analysis of the TCGA‐BLCA bulk RNA‐seq data using CIBERSORTx, estimating the abundance of each fibroblast subpopulations. The proportion of MMP11^+^ mCAFs progressively increased with tumor progression and was predominantly enriched in high‐grade tumors (Figure 2E; Figure S2C, Supporting Information). Validation in three independent GEO datasets (GSE31684, GSE48075, and GSE13507) confirmed a similar trend (Figure 2E).^[^ ^26^, ^27^, ^28^ ^]^ Importantly, higher abundance of MMP11^+^ mCAFs was consistently associated with poorer overall survival across all four datasets (Figure 2F). In contrast, IGLC1^+^ CAFs, another fibroblast subpopulation, exhibited a decreasing trend with tumor progression and were linked to better prognosis (Figure S2D,E, Supporting Information). Notably, a significant negative correlation was observed between MMP11^+^ mCAFs and IGLC1^+^ CAFs across all four datasets (Figure S2F, Supporting Information). These findings suggest that MMP11^+^ mCAFs contribute to tumor progression, while IGLC1^+^ CAFs may be linked to more favorable clinical outcomes.

Further functional analysis of MMP11^+^ mCAFs revealed that DEGs were significantly enriched in pathways related to ECM organization, vasculature development, cell adhesion, BMP and TGFβ1 signaling response (Figure 2G,H). GSEA further demonstrated strong enrichment in hallmark pathway such as epithelial‐mesenchymal transition, angiogenesis, coagulation, and apical junction formation. Collectively, these findings suggest that MMP11^+^ mCAFs may play a key role in promoting tumor angiogenesis (Figure 2I).

### 2.3 MMP11^+^ mCAFs Promote Migration of ESM1^+^ tEC Tip Cells Through WNT5A

**Figure 3:**
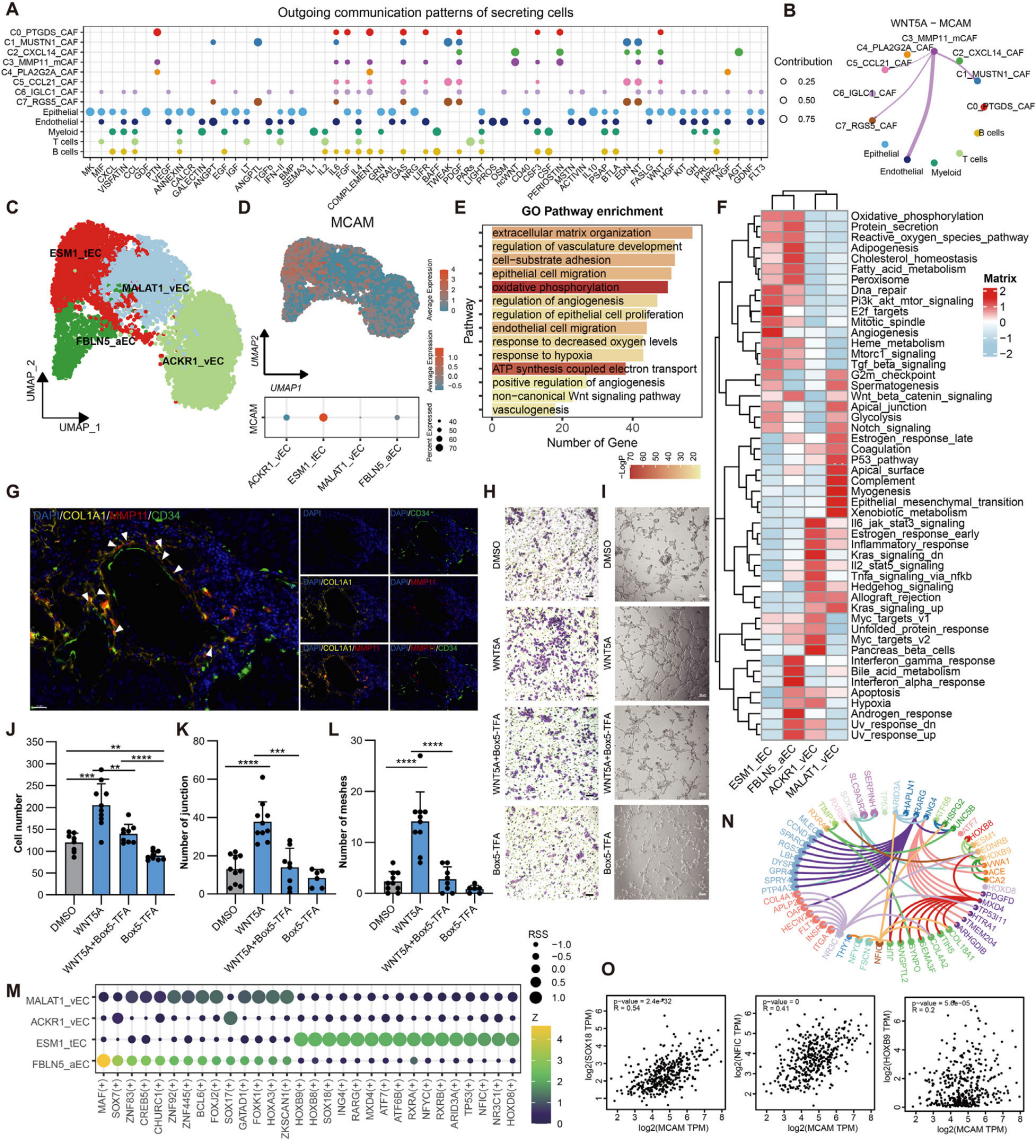
MMP11^+^ mCAFs drive the migration of ESM1^+^ tECs through the WNT5A‐MCAM signaling axis. A) Signaling output landscape of different cell populations, visualized to highlight key pathways and intercellular communication hubs. B) Communication intensity of the WNT5A‐MCAM signaling axis between MMP11^+^ mCAFs with distinct cell populations. C) UMAP plot displaying the classification of endothelial cell subpopulation. D) Expression levels of MCAM across identified endothelial subpopulations, visualized as a UMAP plot (top) and a Dot plot (bottom) overlay. E) GO enrichment analysis showing key biological processes and pathways associated with upregulated genes in ESM1^+^ tECs. F) Heatmap of pathway activity variations scored by GSVA for each cell between different endothelial groups. G) IF staining for spatial localization of MMP11^+^ mCAFs, confirming their proximity to endothelial regions of interest. Scale bar = 20 µm. The white arrow indicates the MMP11^+^ mCAF cells. H) Transwell migration assay demonstrating the effect of WNT5A and Box5‐TFA, with representative images showing cell migration trends. Scale bar = 50 µm, *n* = 3. I) Angiogenesis assays assessing effect of WNT5A and Box5‐TFA on tube formation activity in HUVECs. Scale bar = 50 µm, *n* = 3. J–L) Quantifications of migrated cells in Transwell migration assay (J), junction numbers (K), and mesh numbers (L) in Angiogenesis assays. Statistical analysis was performed using Student's *t*‐test; **p < 0.01, ***p < 0.001, ****p < 0.0001. M) Transcription factors specific to endothelial subpopulations, identified via single‐cell transcriptomics. N) ESM1^+^ tEC‐specific transcription factors and their regulatory target genes, mapped to demonstrate transcriptional control over ESM1^+^ tEC signature genes. O) Correlation between ESM1^+^ tEC‐specific transcription factors genes and *MCAM* expression, tested by Spearman correlation.

To elucidate the mechanisms by which MMP11^+^ mCAFs influence the TME, we conducted comprehensive cell–cell communication analyses across all cell types, identifying the non‐canonical WNT (ncWNT) signaling pathway as particularly active in MMP11^+^ mCAFs (**Figure** **3**A; Figure S3A, Supporting Information). The WNT5A‐MCAM axis emerged as the primary driver of this activity (Figure S3B, Supporting Information), with MMP11^+^ mCAFs exerting a strong influence on endothelial cells through this axis (Figure 3B). To characterize the endothelial compartment, endothelial cells were further isolated and reclustered into four distinct clusters using canonical and cell‐specific marker genes: ACKR1^+^ vEC (venous endothelial cell), MALAT1^+^ vEC, ESM1^+^ tEC (tip endothelial cell), and FBLN5^+^ aEC (arterial endothelial cell) (Figure 3C; Figure S3C–E, Supporting Information).^[^ ^29^ ^]^ Notably, MCAM was predominantly enriched in ESM1^+^ tECs (Figure 3D), suggesting that MMP11^+^ mCAFs may preferentially target this endothelial subset.

Functional analysis of ESM1^+^ tECs revealed significant enrichment in pathways related to ECM remodeling, angiogenesis, endothelial cell migration, hypoxia response, ncWNT signaling, and focal adhesion (Figure 3E; Figure S3F, Supporting Information). Gene set variation analysis (GSVA) further showed that both ESM1^+^ tECs and FBLN5^+^ aECs exhibited enrichment in oxidative phosphorylation, protein secretion, lipogenesis, DNA repair, PI3K‐Akt‐mTOR signaling, and TGF‐β signaling pathways. Importantly, ESM1^+^ tECs displayed stronger associations with angiogenesis‐related programs and E2F target activity, indicating their role in vascular remodeling and development (Figure 3F).

To explore the molecular interactions between MMP11^+^ mCAFs and ESM1^+^ tECs, we applied NicheNet analysis (Figure S3G, Supporting Information). Potential target genes in ESM1^+^ tECs were enriched in biological processes such as vascular development, endothelial cell proliferation, ECM remodeling, and hypoxia response, corroborating the functional characteristics of ESM1^+^ tECs and supporting their pivotal role in angiogenesis (Figure 3E; Figure S3H, Supporting Information).

Accumulating evidence highlights WNT5A and MCAM as key drivers of angiogenesis.^[^ ^30^, ^31^, ^32^, ^33^ ^]^ Given the role of tip endothelial cells in directing angiogenesis through directed migration,^[^ ^34^ ^]^ and the known function of the WNT5A‐MCAM axis in regulating cell motility and convergent extension,^[^ ^35^ ^]^ we hypothesize that MMP11^+^ mCAFs promote the migration of ESM1^+^ tECs via this pathway. IF staining of tumor sections showed that MMP11^+^ mCAFs are spatially adjacent to endothelial cells, providing anatomical evidence for potential paracrine signaling (Figure 3G). Transwell migration and tube formation assays showed that WNT5A significantly enhanced the migratory and tube‐forming abilities of human umbilical vein endothelial cells (HUVECs). Conversely, the WNT5A inhibitor Box5‐TFA impaired both processes, underscoring the importance of WNT5A signaling in endothelial activation (Figure 3H–L). These results suggest that MMP11^+^ mCAFs may facilitate angiogenesis by promoting ESM1^+^ tECs migration through WNT5A signaling.

We further examined the transcription factors active in endothelial cells. ESM1^+^ tECs showed elevated activity of transcription factors including SOX18, several members of HOX and ATF families, as well as RARG, ING4, MXD4, and NFIC (Figure 3M). These factors targeted signature genes specific to ESM1^+^ tECs, with HOXB9 directly regulating *ESM1*, a well‐established angiogenesis‐related gene,^[^ ^36^ ^]^ and SOX18 targeting *EGFL7*, a gene involved in endothelial cell adhesion, cytoskeletal remodeling, and migration.^[^ ^37^ ^]^ NFIC was found to regulate *F* *SCN1*, an actin crosslinker critical for cell migration (Figure 3N).^[^ ^38^, ^39^, ^40^, ^41^ ^]^ Additionally, previous studies have established that SOX18 and HOXB9 are key pro‐angiogenic factors,^[^ ^42^, ^43^, ^44^, ^45^ ^]^ while SOX18, NFIC, and HOXB9 have been implicated in promoting cell migration.^[^ ^46^, ^47^, ^48^, ^49^, ^50^ ^]^

To further explore the upstream regulation of these transcription factors, we examined the relationship between the WNT5A‐MCAM axis and the expression of *SOX18*, *HOXB9*, and *NFIC*. A positive correlation between *MCAM* expression and these transcription factors was observed (Figure 3O). To experimentally validate the role of WNT5A in transcriptional regulation, we treated HUVEC cells with WNT5A and its inhibitor Box5‐TFA in vitro and quantified the expression of *SOX18*, *HOXB9*, and *NFIC*. The results showed that WNT5A stimulation significantly upregulated these genes (Figure S3I–K, Supporting Information). Based on these findings, we speculate that MMP11^+^ mCAFs may contribute to the upregulation of WNT5A, which in turn modulates the expression of specific transcription factors such as *SOX18*, *HOXB9*, and *NFIC* in ESM1^+^ tECs, potentially promoting angiogenesis.

### 2.4 MMP11^+^ mCAFs Mediate SPP1^+^ Macrophage Infiltration and the Production of VEGFA

To explore the association between MMP11⁺ mCAFs and the tumor immune microenvironment, we categorized samples from the TCGA‐BLCA dataset into two distinct molecular subtypes based on the signature gene expression profiles of MMP11⁺ mCAFs (Figure S4A and Table S3, Supporting Information). Subtype 2 exhibited significantly higher stromal and immune scores, ESTIMATE scores, MMP11⁺ mCAF signature scores, and proportions of MMP11⁺ mCAFs compared to Subtype 1, along with notably lower tumor purity (Figure S4B, Supporting Information). Immune infiltration analysis showed increased macrophage infiltration in Subtype 2 (Figure S4C, Supporting Information), prompting further investigation of myeloid cells.

Myeloid cells were isolated and reclustered into monocytes, macrophages, mast cells, cDC1, cDC2, mregDC, and pDC based on canonical and cluster‐specific marker genes (Figure S4D,E, Supporting Information). Notably, macrophages, cDC2, and mregDC were more abundant in tumor tissues compared to normal tissues (Figure S4F, Supporting Information). Cell‐cell communication analysis revealed that MMP11⁺ mCAFs recruit myeloid cells via chemokines, including CCL11, CXCL1, and CXCL6 (Figure S4F, Supporting Information), with the strongest communication occurring between MMP11⁺ mCAFs and monocytes/macrophages, mediated by CXCL12, CCL11, CCL2, and CCL5 (Figure S4G, Supporting Information). This suggests MMP11⁺ mCAFs orchestrate myeloid cell infiltration in the TME.

**Figure 4:**
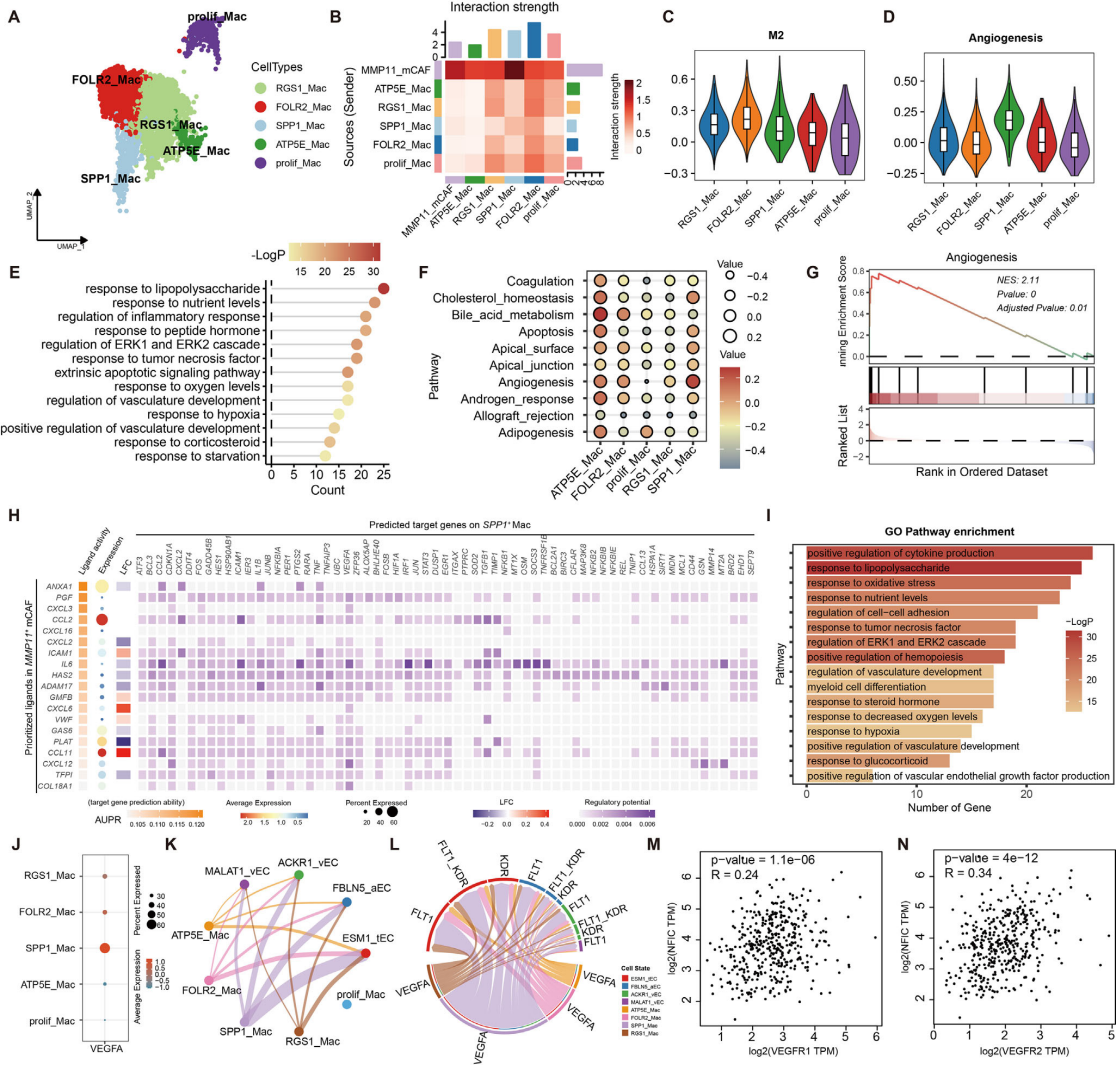
MMP11^+^ mCAFs promote VEGFA production by SPP1^+^ macrophages. A) UMAP plot showing the classification of macrophage subpopulations. B) Interaction strength between MMP11^+^ mCAFs and macrophage subpopulations. C,D) Violin plots showing M2 polarization scores (C) and angiogenesis scores (D) of macrophage subpopulations. E) Enriched GO terms associated with upregulated genes in SPP1^+^ macrophages. F) Heatmap of pathway activity differences across macrophage subpopulations scored by GSVA. G) GSEA of the angiogenesis gene set, ranking genes by log2 fold changes between SPP1^+^ macrophages and other macrophage subpopulations. H) NecheNet analysis illustrating cell‐cell communication between MMP11^+^ mCAFs and SPP1^+^ macrophages, including regulated target genes. I) GO enrichment analysis of genes targeted by MMP11^+^ mCAFs in SPP1^+^ macrophages. J) VEGFA expression levels across macrophage subpopulations. K,L) VEGFA signaling interactions between macrophage subpopulations and endothelial cells, with ligand‐receptor communication strength (K) and receptor distribution (L) visualized. M,N) Spearman correlation between expression of ESM1^+^tEC‐specific transcription factors genes with VEGFA receptor genes *VEGFR1* (M) and *VEGFR2* (N).

Given the observed macrophage infiltration (Figure S4C, Supporting Information), we further reclustered the macrophage into five subgroups: FOLR2^+^ Mac, SPP1^+^ Mac, RGS1^+^ Mac, ATP5E^+^ Mac, and prolif Mac (**Figure** **4**A; Figure S4H, Supporting Information). The strongest interactions between MMP11⁺ mCAFs and macrophages were observed in the SPP1^+^ Mac subgroup (Figure 4B). Notably, SPP1^+^ Mac, along with RGS1^+^ Mac and FOLR2^+^ Mac, underwent M2 polarization, with SPP1^+^ Mac exhibiting the highest angiogenesis scores (Figure 4C,D). Functional enrichment analysis revealed that SPP1⁺ Mac was significantly involved in cellular signaling and vascular development (Figure 4E–G), highlighting their role in promoting tumor angiogenesis through enhanced pro‐angiogenic signaling.

To explore the communication between MMP11⁺ mCAFs and SPP1^+^ Mac, we used NicheNet to identify target genes regulated by MMP11⁺ mCAFs in SPP1^+^ Mac (Figure 4H). This analysis revealed that MMP11⁺ mCAFs influence SPP1^+^ Mac through multiple signaling pathways, with ANXA1 emerging as a key regulator of cytokines such as CCL2, CXCL2, IL1B, and TNF. Among the ligands, CCL2 and CCL11 were the most prominently expressed (Figure 4H). Gene Ontology (GO) enrichment of the target genes highlighted their involvement in cytokine production, nutrient response, hypoxia adaptation, and angiogenesis (Figure 4I). Moreover, a specific GO term related to the positive regulation of VEGFA production, a critical angiogenesis mediator, was identified (Figure 4I). NicheNet analysis confirmed that CCL2 and CCL11 act as inducers of *VEGFA* expression (Figure 4H). Expression analysis showed that *VEGFA* was predominantly expressed in SPP1^+^ Mac (Figure 4J), leading us to hypothesize that MMP11⁺ mCAFs promote *VEGFA* production in SPP1^+^ Mac through these signaling pathways, thereby facilitating angiogenesis.

Further communication analysis between macrophages and endothelial cells revealed that VEGFA signaling, primarily originating from SPP1^+^ Mac, targets ESM1^+^ tECs (Figure 4K). This interaction is mediated by VEGFR1 and VEGFR2, which regulate ESM1^+^ tEC function (Figure 4L). Correlation analysis showed that VEGFA signaling was positively associated with the expression of *NFIC* (Figure 4M,N). In summary, we propose that MMP11⁺ mCAFs recruit and activate SPP1^+^ Mac to upregulate VEGFA production, which in turn influences ESM1^+^ tECs and promotes angiogenesis.

### 2.5 Interferon‐Associated Basal‐Like Tumor Cells Secrete BMP2 to Induce the Expression of Characteristic Genes in MMP11^+^ mCAFs

**Figure 5:**
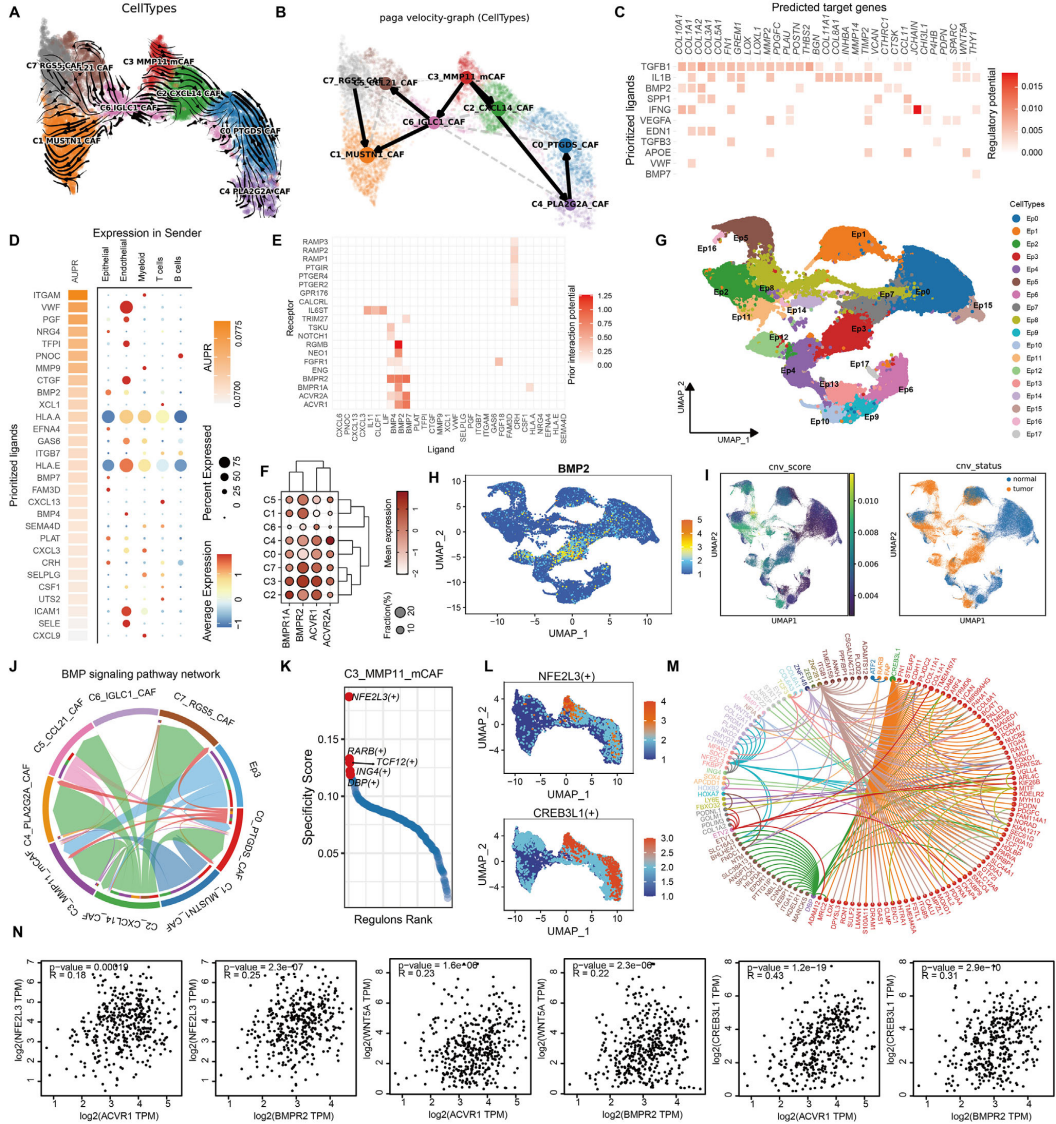
Tumor‐secreted BMP2 induces the expression of MMP11^+^ mCAF signature genes. A,B) RNA velocity analysis (A) and PAGA (partition‐based graph abstraction) analysis (B) of fibroblasts. C) NechNet analysis showing cytokine signaling that regulates highly dynamic genes in MMP11^+^ mCAFs. D) NechNet analysis showing ligand activity (left) and ligand expression in different cell populations (right), with MMP11^+^ mCAFs as the receptor cell population. E) NechNet analysis display receptor activity in MMP11^+^ mCAFs. F) Expression levels of BMP receptor genes across fibroblast subpopulations. G) UMAP plot showing epithelial cell classification. H) UMAP plot showing the expression distribution of BMP2 in epithelial cells. I) Identification of normal epithelial cells and tumor cells, with CNV_score > 0.005 considered indicative of tumor cells. J) Interaction strength of the BMP signaling pathway network between Ep3 and fibroblast subclusters. K) Key transcription factors in MMP11^+^ mCAFs. L) UMAP plot showing the activity distribution of MMP11^+^ mCAF‐specific transcription factors across fibroblast subpopulations. M) Target genes regulated by MMP11^+^ mCAF‐specific transcription factors. N) Spearman correlation analysis showing significant positive correlations between MMP11^+^ mCAF‐specific transcription factors, signature genes, and BMP2 receptor gene expression.

To investigate the origin of MMP11⁺ mCAFs, we performed RNA velocity analysis across all fibroblast populations. The results indicated that MMP11⁺ mCAFs are at the initiation point of differentiation (**Figure** **5**A; Figure S5A–C, Supporting Information). PAGA analysis revealed three differentiation trajectories: MMP11⁺ mCAFs transitioning into IGLC1^+^ CAFs, CXCL14^+^ CAFs, and progressing through PLA2G2A^+^ CAFs to PTGDS^+^ CAFs (Figure 5B). Given the progressive increase in MMP11⁺ mCAFs with tumor development, we hypothesize that this population may revert along these pathways. Further confirmation from Slingshot, Cytotrace, and Monocle3 analysis supports that MMP11⁺ mCAFs represent a low‐differentiated state (Figure S5D–I, Supporting Information). Pseudotime trajectory analysis of MMP11⁺ mCAFs showed significant enrichment in gene modules related to ECM remodeling, collagen metabolism, endothelial cell proliferation, BMP binding, and immune system regulation (Figure S5J–L, Supporting Information).

We constructed a gene set based on MMP11⁺ mCAF signature genes reflecting RNA velocity dynamics and identified TGFB1, IL1B, BMP2, and SPP1 signaling as key modulators (Figure 5C). Further investigation revealed that BMP2, primarily secreted by epithelial cells, regulates MMP11⁺ mCAFs in the TME (Figure 5D; Figure S7A, Supporting Information). Additionally, MMP11⁺ mCAFs exhibited relatively high BMP signaling receptor activity (Figure 5E,F), suggesting BMP2 signaling drives their formation.

To explore the expression of *BMP2* in epithelial cells, epithelial cells were reclustered into 18 distinct clusters, with *BMP2* predominantly expressed in the Ep3 cluster, which is largely tumor‐derived (Figure 5G,H; Figure S6A, Supporting Information). Copy number variation (CNV) analysis confirmed that the Ep3 cluster mainly composed of tumor cells (Figure 5I; Figure S6B, Supporting Information). Tumor cell characterization revealed that Ep3 cells exhibit high expression of interferon‐related genes (*IFI27*, *IFI6*) and are involved in antiviral immune responses, cellular stress, immune regulation, and cholesterol homeostasis (Figure S6C–F, Supporting Information). Further analysis identified Basal, Stress, Hypoxia, pEMT, Interferon, and Oxphos signatures in Ep3 cells (Figure S6G, Supporting Information),^[^ ^51^ ^]^ classifying them as interferon‐associated basal‐like tumor cells (IFN‐BL tumor cells). CellPhoneDB analysis revealed strong interactions between Ep3 and MMP11⁺ mCAFs (Figure S6H, Supporting Information), while CellChat analysis indicated that BMP signaling from Ep3 primarily targets MMP11⁺ mCAFs (Figure 5J). These findings support the hypothesis that IFN‐BL tumor cells secrete BMP2, driving the formation of MMP11⁺ mCAFs.

We next analyzed the transcription factors within fibroblast clusters, identifying NFE2L3, RARB, TCF12, and ING4 as the most specific transcription factors in MMP11⁺ mCAFs (Figure 5K,L; Figure S7B,C, Supporting Information). CREB3L1 ranked highly in terms of activity (Figure 5L). Analysis of the top 15 transcription factors with the greatest activity or specificity revealed that CREB3L1 regulates most MMP11⁺ mCAF‐specific genes, while NFE2L3 primarily regulates *WNT5A* (Figure 5M). In TCGA‐BLCA samples, *NFE2L3*, *CREB3L1*, and *WNT5A* expression was positive correlated with BMP signaling receptors *ACVR1* and *BMPR2* (Figure 5N; Figure S7D, Supporting Information). Additionally, in MMP11⁺ mCAFs, NFE2L3 activity correlated with BMP receptor expression and *WNT5A* (Figure S7E, Supporting Information). In vitro quantitative experiments demonstrated that BMP2 treatment induced the expression of transcription factors *NFE2L3* and *CREB3L1*, as well as several signature genes of MMP11⁺ CAFs, including *WNT5A*, *MMP11*, and *POSTN* (Figure S7F–J, Supporting Information). These results suggest that IFN‐BL tumor cells may promote the expression and activity of CREB3L1 and NFE2L3 in MMP11⁺ mCAFs via BMP signaling, thereby inducing the expression of MMP11⁺ mCAF signature genes.

### 2.6 BMP2 Promotes Tumor Growth and Angiogenesis in a Mouse Bladder Cancer Model

**Figure 6:**
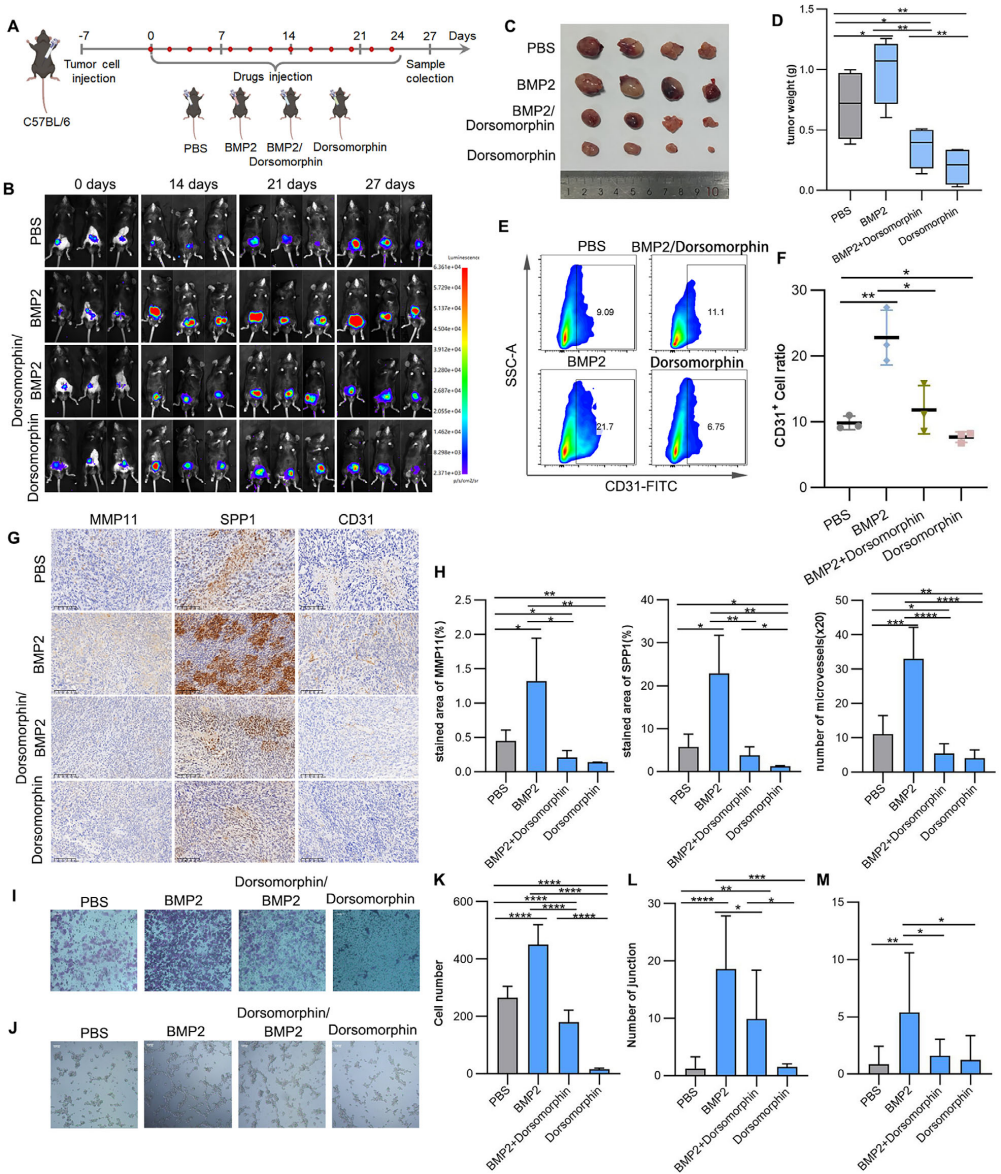
BMP2 promotes tumor growth and angiogenesis in mice. A) Schematic representation of the in vivo biodistribution experiment in tumor‐bearing mice. B) Bioluminescence imaging of tumors in live mice. C) Representative tumor images. D) Tumor volume measurement at the experimental endpoint (*n* = 4). E,F) Flow cytometry analysis of endothelial cell content in tumors (*n* = 3). E) Representative endothelial cell density images in tumor tissues. F) Quantification of endothelial cell content in tumors across different treatment groups. G) IHC analysis showing the expression of MMP11^+^, SPP1^+^, and CD31^+^ cells in tumors from different treatment groups. H) Quantification the stained area of MMP11 and SPP1 in the IHC experiment (*n* = 4) and the number of CD31‐labeled microvessels under 20x magnification (*n* = 8). I,J) Representative images of Transwell migration (I) and tube formation (J) assays of HUVEC treated with conditioned media derived from HBdSFs after treatment with BMP2 or the BMP pathway inhibitor Dorsomorphin. K) Quantification of cell migration in the Transwell assay (I). L,M) Quantification of junction and mesh formation in the tube formation assay (J). Data are presented as mean ± SD. Statistical analysis was performed using Student's *t*‐test; *p < 0.05; **p < 0.01; ***p < 0.001; ****p < 0.0001.

To investigate the role of BMP2 in bladder cancer progression, we treated C57BL/6J mouse bladder orthotopic tumor models with BMP2 and its small molecule inhibitor, Dorsomorphin. BMP2 treatment significantly increased tumor burden compared to the PBS control group, as evidenced by a higher tumor weight at the experimental endpoint (**Figure** **6**A–D). In contrast, inhibition of BMP signaling with Dorsomorphin mitigated tumor progression (Figure 6A–D), indicating that BMP2 enhances tumor growth in vivo.

Flow cytometry analysis further revealed a marked increase in vascular endothelial cells in the BMP2‐treated group compared to controls, whereas Dorsomorphin treatment reduced their abundance (Figure 6E,F), suggesting that BMP2 promotes angiogenesis within the TME. Immunohistochemical (IHC) and IF analysis revealed a marked enrichment of MMP11^+^, SPP1^+^, and CD31^+^ cells in BMP2‐treated tumors, with the opposite pattern observed in the inhibitor‐treated group (Figure 6G,H; Figure S8A,B, Supporting Information). SPP1^+^ cells displayed clear clustering in both PBS and BMP2‐treated groups, while CD31^+^ endothelial cells in BMP2‐treated tumors formed denser and more intricate vascular structures.

Given that BMP2 may induce the expression of *WNT5A* (Figure 5M,N; Figure S7E,H, Supporting Information), which is known to promote endothelial cell migration and tube formation, we conducted conditioned medium transfer experiments. Fibroblast cells (HBdSF) were treated with BMP2 as well as with the BMP pathway inhibitors Dorsomorphin and LDN193189. The resulting conditioned medium was then transferred to HUVECs. Conditioned medium from BMP2‐treated HBdSFs significantly enhanced HUVEC migration and tube formation, whereas inhibitors markedly suppressed these processes (Figure 6I–M; Figure S8C–H, Supporting Information). In conclusion, our results demonstrate that BMP2 enhances tumor growth and angiogenesis in a mouse bladder cancer model, likely through the induction of *WNT5A* and modulation of endothelial cell behavior.

### 2.7 MMP11^+^ mCAFs are Widely Present in Various Cancers

**Figure 7:**
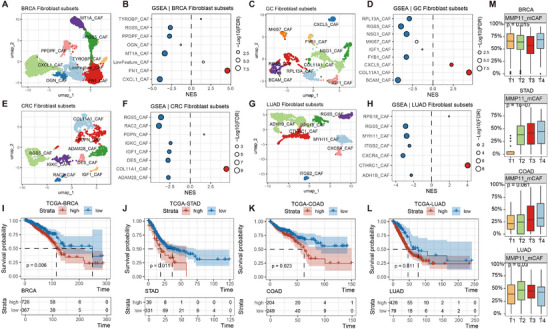
Pan‐cancer analysis of MMP11^+^ mCAFs. A,C,E,G) UMAP plots showing fibroblast subpopulation identification in breast cancer (A), gastric cancer (C), colorectal cancer (E), and lung adenocarcinoma (G). B,D,F,H) GSEA on genes ranked by log2FC between each fibroblast subset and other fibroblasts in breast cancer (B), gastric cancer (D), colorectal cancer (F), and lung adenocarcinoma (H), using MMP11^+^ mCAF signature genes. NES: Normalized Enrichment Score. I–L) Kaplan–Meier survival curves showing overall survival of patients stratified by high or low proportions of MMP11^+^ mCAFs in breast cancer (I), gastric cancer (J), colorectal cancer (K), and lung adenocarcinoma (L). Statistical significance was determined by the log‐rank test. M) Proportion of MMP11^+^ mCAFs across clinical stages in the four cancer types. Statistical analysis was performed using the Kruskal test.

To investigate the presence of MMP11^+^ mCAFs across various cancer types, we performed a pan‐cancer analysis. Fibroblasts from breast cancer, gastric cancer, colorectal cancer, and lung adenocarcinoma were subjected to dimensionality reduction and clustering (**Figure** **7**A,C,E,G). Using the signature genes of MMP11^+^ mCAFs, we constructed a gene set for GSEA analysis. The results revealed that MMP11^+^ mCAFs predominantly overlapped with FN1^+^ CAFs in breast cancer, COL11A1^+^ CAFs in colorectal cancer, and CTHRC1^+^ CAFs in lung adenocarcinoma. In gastric cancer, MMP11^+^ mCAFs were primarily associated with COL11A^+^ CAFs, with a secondary association to CXCL5^+^ CAFs (Figure 7B,D,F,H).

We further applied the CIBERSORT deconvolution algorithm to estimate the proportion of MMP11^+^ CAFs in TCGA datasets corresponding to these cancers and explored their relationship with tumor progression. Analysis revealed that the proportion of MMP11^+^ mCAFs varied significantly across different stages of gastric cancer, breast cancer, and lung adenocarcinoma. In gastric and colorectal cancers, the prevalence of MMP11+ mCAFs increased with tumor progression, although stage‐specific differences in colorectal cancer were not statistically significant (Figure 7M). Despite this variability, survival analysis indicated that MMP11^+^ mCAFs were consistently associated with poor prognosis across all four cancer types (Figure 7I–L). These findings suggest that while MMP11^+^ mCAFs are linked to tumor progression in certain cancers, they are generally associated with poor clinical outcomes across multiple malignancies, highlighting their potential as a universal therapeutic target in cancer treatment.

## 3 Discussion

In this study, we identified a prognostically unfavorable fibroblast subpopulation associated with tumor progression, characterized by high expression of *MMP11*, which we designated as MMP11^+^ mCAF. A similar *MMP11*‐expressing myofibroblast cluster, associated with ECM remodeling, has previously been identified in ovarian cancer.^[^ ^52^ ^]^ This population is induced by TGF‐β and expresses markers including *ACTA2*, *POSTN*, *COL10A1*, *MMP11*, and *TAGLN*. Comparative analysis of the two populations reveals significant overlap in marker gene expression, suggesting that our MMP11+ mCAF subpopulation shares several features with the previously identified myofibroblast cluster, despite some differences in marker profiles. Both subpopulations are enriched in genes involved in ECM remodeling. The observed discrepancies in marker expression may reflect tumor type‐specific differences, tissue origin variations, or methodological differences in clustering approaches, which could contribute to the heterogeneity between these populations.

WNT5A plays a multifaceted role in cancer. Previous research by Zikun Ma et al. demonstrated that a subpopulation of bladder CAFs (SLC14A1^+^ CAFs), promotes cancer stemness in bladder cancer cells via the WNT5A signaling pathway, thereby contributing to tumor aggressiveness and chemotherapy resistance.^[^ ^25^ ^]^ In this study, we identified a distinct mechanism whereby MMP11^+^ mCAFs regulate the migration of ESM1^+^ tEC through a WNT5A‐mediated ncWNT signaling axis. Previous research has shown that WNT5A utilizes MCAM (CD146) as its receptor to regulate cellular movement and convergent extension.^[^ ^35^ ^]^ GO enrichment analysis further supports this, revealing that ESM1+ tECs are enriched in genes related to ncWNT signaling and endothelial cell migration. Functional assays confirmed that WNT5A promotes the migration of HUVECs. Based on the expression of specific cell markers, we identified the ESM1^+^ tEC population as tip cells within the context of angiogenesis.^[^ ^29^ ^]^ Tip cells play a pivotal role in blood vessel formation, acting as “leaders” by migrating and sensing the surrounding chemical gradients (e.g., VEGFA) to guide the direction of vascular growth.^[^ ^34^ ^]^ The ESM1^+^ endothelial cell population has also been implicated in promoting angiogenesis in other contexts. For instance, in gastric cancer, fibroblast subpopulations have been shown to interact with EC‐ESM (tip‐like endothelial) cells to enhance blood vessel formation.^[^ ^53^ ^]^ Taken together, our data suggest that the MMP11^+^ mCAF subpopulation may enhances the migratory capacity of the ESM^+^ tEC cells via the WNT5A‐MCAM signaling axis, thereby potentially facilitating angiogenesis. From the perspective of angiogenesis, our findings provide deeper insights into how CAFs may exert a paradoxical role in promoting tumor progression, simultaneously contributing to both tumorigenesis and the vascularization of tumors.

In addition, our study reveals that the MMP11^+^ mCAF subpopulation may not only directly interacts with endothelial cells but could also exerts an indirect effect through the recruitment of SPP1^+^ Mac, promoting their production of VEGFA, which further influences endothelial cells. Previous research by Zemin Zhang's group demonstrated that SPP1^+^ tumor‐associated macrophages (TAMs) primarily function to promote angiogenesis and facilitate tumor metastasis.^[^ ^54^ ^]^ Similarly, Xu Pan et al. reported that SPP1^+^ TAMs frequently interact with endothelial cells, with a particularly close spatial relationship with tip cells.^[^ ^55^ ^]^ In our study, we observed that MMP11^+^ mCAFs exhibit the strongest interaction with SPP1^+^ Mac among all macrophage subpopulations, and that this interaction enhances VEGFA production by SPP1^+^ Mac. Notably, the VEGFA secreted by SPP1^+^ Mac predominantly targets tip cells. Previous studies have established that VEGFA is essential for the formation of tip cells and the extension of filopodia, which are crucial for the migration of endothelial cells.^[^ ^56^, ^57^, ^58^, ^59^ ^]^ Taken together, our findings suggest that MMP11^+^ mCAFs can induce the formation and migration of tip endothelial cells both directly and indirectly, thereby contributing to tumor angiogenesis. These results highlight the complex interplay between fibroblasts, macrophages, and endothelial cells in promoting vascularization within the TME, further emphasizing the role of MMP11^+^ mCAFs in facilitating tumor progression.

Mechanistically, CAFs exist in a dynamic and transient state, regulated by a variety of factors through both autocrine and paracrine signaling, granting them remarkable plasticity. For instance, TGF‐β family ligands are key drivers of fibroblast activation, with TGFB1 considered a primary stimulator of myofibroblast differentiation.^[^ ^60^, ^61^, ^62^, ^63^ ^]^ Conversely, IL‐1 is a known inducer of inflammatory fibroblasts.^[^ ^9^, ^64^ ^]^ Therefore, selective elimination or remodeling of specific CAFs subpopulations, or inhibition of downstream signaling pathways in CAFs, may represent effective therapeutic strategies in cancer treatment. In our study, MMP11^+^ mCAFs are characterized by signature genes and functional pathways that align them with the myofibroblast lineage. These cells are not only activated by TGF‐β signaling but also exhibit gene signatures responsive to BMP2. Previous research has suggested that BMP2 and BMP4 can induce the transdifferentiation of myofibroblasts into adipocyte‐like cells that store lipids.^[^ ^65^ ^]^ In pancreatic cancer, for example, tumor cells have been shown to secrete BMP2, promoting the differentiation of lipid‐rich CAFs.^[^ ^66^ ^]^ In this context, we found that BMP2 may play a role in promoting the transformation of cancer‐associated myofibroblasts into the MMP11^+^ mCAFs subpopulation. This suggests that targeting BMP2 could potentially reprogram the MMP11^+^ mCAFs population.

In our experimental models, we employed the BMP2 inhibitor Dorsomorphin in mice with bladder tumors and observed a significant inhibition of tumor progression. Targeting BMP signaling has also been shown to suppress tumor metastasis in pancreatic cancer.^[^ ^66^ ^]^ Additionally, another study demonstrated that BMP2 promotes melanoma growth, and its inhibition sensitizes tumors to therapeutic treatments.^[^ ^67^ ^]^ A similar effect has been reported in ovarian cancer, where BMP2 inhibition not only suppresses tumor growth but also reverses chemotherapy resistance.^[^ ^68^ ^]^ These findings suggest that targeting BMP2 could offer a promising therapeutic approach across various cancer types, including those with poor prognostic features, such as tumors enriched in MMP11^+^ mCAFs. Notably, we have identified similar MMP11^+^ mCAFs populations in other cancer types (Figure 7), which are also associated with poor prognosis. This underscores the clinical relevance of exploring targeted therapies aimed at these cells. Further investigation into combining BMP2 inhibition with other therapeutic strategies, such as immunotherapy, may provide a more comprehensive approach to improving cancer treatment outcomes.

An intriguing finding in our study is the observation that, during tumor progression, the MMP11^+^ mCAFs subpopulation exhibits an inverse trend compared to the IGLC1^+^ CAFs subpopulation, which is associated with a better prognosis. This inverse relationship between the two fibroblast populations is consistently observed across multiple datasets (Figure S2F, Supporting information). Furthermore, RNA velocity analysis reveals a dynamic transition between MMP11^+^ mCAFs and other fibroblast subpopulations, including IGLC1^+^ CAFs (Figure 5A). Notably, in the later stages of tumor progression, the poor‐prognosis MMP11^+^ mCAFs predominate, whereas IGLC1^+^ CAFs are more prevalent in the early stages. These findings suggest that the transition between these two fibroblast states may play a pivotal role in tumor progression and prognosis. Investigating the molecular mechanisms underlying this cellular state transition could provide valuable insights into the evolving TME. Moreover, strategies aimed at reversing the transition from the poor‐prognosis MMP11^+^ mCAFs state to the more favorable IGLC1^+^ CAFs state could offer a novel and potentially more effective therapeutic approach. Targeting these specific fibroblast populations and modulating their differentiation or functional state may improve patient outcomes and enhance the efficacy of cancer treatments.

Despite the valuable insights provided by our study, several limitations should be acknowledged. While Dorsomorphin treatment reduced MMP11⁺ mCAFs and suppressed tumor angiogenesis, its antitumor effects may also involve other mechanisms, such as reducing HSF1 activity or inhibiting ABCG2 function, as previously reported.^[^ ^69^, ^70^ ^]^ Thus, attributing its effects solely to BMP2 inhibition in MMP11⁺ mCAFs remains challenging, and potential off‐target effects warrant further investigation. Beyond pharmacological concerns, some mechanistic insights—particularly the direct and indirect roles of MMP11⁺ mCAFs in angiogenesis—were primarily based on bioinformatics analyses and in vitro simulations. Although tube formation and conditioned medium experiments support their pro‐angiogenic function, further validation using co‐culture systems or in vivo models is needed. Future studies should consider genetic perturbation strategies, such as CRISPR/Cas9‐mediated knockout or RNA interference, combined with lineage tracing, to more precisely delineate the role of BMP2 signaling in MMP11⁺ mCAF‐mediated tumor progression. Additionally, identifying more selective BMP2 inhibitors or optimizing Dorsomorphin dosing may help reduce off‐target effects and improve therapeutic potential.

## 4 Conclusion 

This study identified a new subgroup of CAFs characterized as MMP11^+^ mCAFs, which increases progressively with the advancement of bladder cancer and is associated with poor prognosis. The findings elucidate the mechanisms through which this CAF population promotes tumor angiogenesis by regulating tip endothelial cell migration through both direct and indirect pathways. Furthermore, the study revealed the widespread presence of MMP11^+^ mCAFs across multiple cancer types, offering a novel potential therapeutic target for cancer treatment.

## 5 Experimental Section

### Patients and Samples

In this study, the in‐house single‐cell dataset was integrated with two publicly available datasets.^[^ ^23^, ^24^ ^]^ The inclusion criteria were patients’ individuals diagnosed with bladder cancer, aged 25–85 years, with pathological stages ranging from T0 to T4, and no prior exposure to chemotherapy or radiotherapy. The combined dataset comprised 22 tumor samples and 7 adjacent normal tissue samples, spanning pathological stages T0 to T3. Clinical metadata for the public datasets were obtained from the original publications, while comprehensive clinical details for the in‐house dataset are provided in Table S2 (Supporting Information). The internal bladder samples were obtained from patients undergoing transurethral resection of bladder tumors at Shenzhen Luohu District People's Hospital. The experimental procedures were approved by the Institutional Review Board (IRB) of Shenzhen Luohu District People's Hospital (IRB approval number: 2023‐GDSKJ‐07). Fresh tumor and adjacent normal mucosal samples were collected, rapidly rinsed with saline, and then preserved in GEXSCOPETM Tissue Preservation Solution (Singleron, Cologne, Germany). Subsequently, the samples were cut into <2 mm pieces, digested with tissue dissociation solution at 37 °C for 15 min and filtered through a sterile 40 µm mesh. All cells were collected by centrifugation at 250 g for 5 min at 4 °C and washed with PBS buffer. Red blood cells were lysed by incubating with 2 mL GEXSCOPETM red blood cell lysis buffer (Singleron, Cologne, Germany) at 4 °C for 10 min. The cells were then washed with 10 mL PBS buffer, centrifuged again at 250 g for 10 min, and resuspended in PBS buffer for further analysis.

Publicly available single‐cell datasets used in this study can be accessed from the following sources: BioProject PRJNA662018 in the SRA database, GEO datasets GSE135337, GSE161529 (BRCA), GSE188711 (CRC), and GSE163558 (GC). The LUAD single‐cell dataset is available at https://doi.org/10.24433/CO.0121060.v1. The Affymetrix microarray datasets used in this study include GSE31684, GSE48075, and GSE13507. Furthermore, TCGA datasets for BLCA, BRCA, STAD, COAD, and LUAD were obtained from UCSC Xena (http://xena.ucsc.edu/).

### Single‐Cell RNA Sequencing and Raw Data Processing

Single‐cell suspensions were captured using the GEXSCOPER microfluidic chip according to the Singleron GEXSCOPER protocol. Libraries were constructed using the GEXSCOPER Single‐Cell RNA Library Kit (Singleron Biotechnologies, Nanjing, China) and sequenced on the Illumina HiSeq X platform (Illumina, USA) with 150 base pair paired‐end reads. The raw data was processed using the Celescope pipeline (v1.7.2, https://github.com/singleron‐RD/CeleScope) developed by Singleron. Quality control and data filtering were performed using FASTQC (version 0.11.7) and Cutadapt (version 1.17). Sequence reads were aligned to the GRCh38 reference genome using STAR (version 2.6.1b). Gene expression matrices for downstream analyses were generated using FeatureCounts (version 1.6.2).

### Dimensionality Reduction, Clustering, and Cell Group Annotation

To analyze the single‐cell transcriptomic data, Seurat (v4.3.0) was employed for processing both the own dataset and two publicly available datasets. Cells expressing fewer than 200 genes or with mitochondrial UMI percentages exceeding 10% were excluded from downstream analyses. All single‐cell datasets were subsequently integrated, and the UMI count matrix was subjected to standardization and normalization following Seurat's established pipeline. Principal component analysis (PCA) was performed using the RunPCA function in Seurat, and batch effects among samples were addressed using Harmony (v0.1.1).

Clustering was conducted using the FindClusters function in Seurat across multiple resolutions, followed by dimensionality reduction with UMAP using the top 15 principal components through the RunUMAP function. DEGs between clusters were identified using the FindAllMarkers function with the parameters min.pct = 0.25 and logfc.threshold = 0.75. DEG significance was assessed using the non‐parametric Wilcoxon rank‐sum test, with *p*‐values adjusted via Bonferroni correction. An adjusted *p*‐value < 0.05 was considered statistically significant. Cluster annotation was performed manually based on canonical cell‐type markers and cluster‐specific marker genes.

For each major cell type, further normalization, dimensionality reduction, batch effect correction with Harmony, and clustering were performed. Subclusters co‐expressing marker genes of two or more major cell types were manually identified and excluded as doublets. The remaining cells were re‐analyzed using the aforementioned pipeline to define major cell types and subclusters.

### Functional Enrichment Analysis

To assess functional enrichment, gene set scores were assigned to each cell type using the AddModuleScore function in the Seurat package. DEGs between two cell populations were identified using the FindMarkers function. GO, Kyoto Encyclopedia of Genes and Genomes (KEGG), and GSEA were performed using the ClusterProfiler package (v3.18.1).^[^ ^71^ ^]^ To evaluate differences in pathway activity among cell types, GSVA was conducted using the GSVA package (v1.30.0) with default parameters.^[^ ^72^ ^]^

### Single‐Cell Trajectory Analysis

To investigate the differentiation trajectory of fibroblasts, RNA velocity analysis, slingshot, and Monocle3 were applied. RNA velocity estimation was performed using the scVelo package (v0.2.5, https://github.com/theislab/scvelo),^[^ ^73^, ^74^ ^]^ implemented in Python v3.7.12 within the Conda environment (v23.7.4). Spliced and unspliced mRNA count matrices were first derived from BAM files and converted into loom format. Loom files generated by Velocyto were subsequently imported, and cell barcodes were standardized for consistency. The datasets were integrated using scVelo to quantify the proportions of spliced, unspliced, and ambiguous RNA across cell types. RNA velocity was calculated in stochastic mode, and velocity vectors were projected onto UMAP embeddings to visualize dynamic transitions. Downstream analyses involved the identification of genes driving RNA velocity, pseudotime inference, and trajectory reconstruction using PAGA. The results were represented as velocity streamlines and lineage graphs, providing insights into cellular transitions and connectivity. Furthermore, spatial dynamics were explored using Monocle to project the velocity vectors and highlight transitions across pseudotime.

Slingshot (v2.7.0) was applied to infer differentiation trajectories. The Seurat object was converted into a SingleCellExperiment object, and UMAP was used for dimensionality reduction and clustering based on cell type annotations. The inferred trajectory, approximated using 150 points, was visualized by overlaying pseudotime values and trajectory paths on UMAP embeddings.

For Monocle3 (v1.3.1), a CellDataSet object was constructed from the gene expression matrix and cell metadata extracted from the Seurat object, incorporating both cell and gene annotation information. Data normalization and selection of highly variable genes were performed, followed by dimensionality reduction and clustering. Trajectories were reconstructed using the learn_graph function, and pseudotime ordering was applied based on the trajectory graph. DEGs along the pseudotime trajectory were identified using the graph_test function, with a *q*‐value threshold of < 0.05. Co‐expressed gene modules related to differentiation were identified using the find_gene_modules function, and their expression patterns were visualized via heatmaps across different cell populations.

### CytoTRACE Analysis for Fibroblast Differentiation Assessment

To assess the differentiation status of fibroblasts, CytoTRACE analysis (v0.3.3) was employed. First, the gene expression matrix was extracted from the single‐cell RNA sequencing dataset and filtered out genes with low expression, retaining only those expressed in at least five cells. The filtered expression matrix was then used as input for the CytoTRACE function, which estimates the differentiation potential of individual cells. CytoTRACE scores range from 0 to 1, with higher scores indicating stronger stemness, and lower scores reflecting a higher degree of differentiation.

### Estimation of CNVs in Cancer Cells

Chromosomal copy number variations (CNVs) were analyzed using the inferCNV framework,^[^ ^75^ ^]^ implemented with infercnvpy (v0.4.5, https://github.com/icbi‐lab/infercnvpy). In brief, CNV profiles were inferred by mapping gene‐level expression data to their corresponding chromosomal locations. After filtering out genes lacking chromosomal information, CNVs were inferred using cells from normal tissue as the reference. Clustering of cells was performed based on their CNV profiles, followed by UMAP visualization to explore CNV patterns. Tumor cells were identified based on a CNV score threshold of >0.005. Chromosomal CNV heatmaps and UMAP plots colored by CNV status were generated for visualization.

### Simultaneous Gene Regulatory Network Analysis

Single‐cell transcription factor (TF) activity was analyzed using the pySCENIC package (v0.12.1, https://github.com/aertslab/pySCENIC) in a Python environment (Python v3.7.12, conda v23.9.0).^[^ ^76^ ^]^ TF annotations and motif databases were obtained from recommended sources. The expression matrix was converted into a loom file, ensuring compatibility with pySCENIC's downstream analyses. Gene regulatory networks (GRNs) were inferred using the GRNBoost2 algorithm with default parameters applied to the loom file. Subsequently, the inferred networks were contextualized using the ctx function, which integrates motif enrichment analysis and module annotation. This step was performed in a distributed manner with Dask multiprocessing, utilizing TF motif data and gene‐to‐motif ranking files, resulting in a regulon activity file. Finally, cell‐specific TF activity scores were quantified using the AUCell method, and the results were exported as a loom file.

### Cell Communication Analysis

The analysis of cell communication was performed using the CellChat (v1.6.1, https://github.com/sqjin/CellChat), NicheNet (v2.0.2, https://github.com/saeyslab/nichenetr) R packages and CellphoneDB (v3.1.0, https://github.com/Teichlab/cellphonedb) Python package on python 3.7.12 platform with their default parameters.^[^ ^77^, ^78^, ^79^ ^]^ Input data for CellChat were extracted from Seurat objects, and communication probabilities between different cell groups were calculated using the computeCommunProb function in CellChat. The inferred cell‐cell communication networks were visualized using the netAnalysis_dot function to present the output communication patterns of specific cell groups and either netVisual_bubble or netVisual_aggregate to display specific signaling pathways. The plotGeneExpression function was used to visualize the expression of signaling‐related genes.

The NicheNet package was employed to analyze ligand activity, receptor interaction, and potential target genes between sender and receiver cell groups. For receiver cells, the nichenet_seuratobj_aggregate function was used to compute ligand‐receptor activity and identify potential target genes with the following thresholds: expression_pct = 0.10, lfc_cutoff = 0.25, and cutoff_visualization = 0.33. Alternatively, the highly dynamic gene set identified from MMP11^+^ mCAFs in RNA velocity analysis was used as the target, and ligand activities were predicted using the predict_ligand_activities function. After calculating the differential expression and activity of ligands among sender cells, the connections between ligands‐receptors and ligands‐targets were prioritized. The top‐ranked ligands in the sender cell groups were selected, and only those target genes with scores exceeding 0.25 were retained. Additionally, cell‐cell communication strength between the Ep3 epithelial cell cluster and the fibroblast cluster was inferred using the default parameters of CellphoneDB, and the results were visualized in a heatmap.

### Deconvolution Analysis

Deconvolution analysis was performed to estimate the composition of different cell types using the digital cytometry tool CIBERSORTx (https://cibersortx.stanford.edu/).^[^ ^80^ ^]^ CIBERSORTx leverages gene expression data alongside known associations between genes and specific cell types to infer the relative abundance of various cell populations within heterogeneous samples. For this analysis, the expression matrix of fibroblasts extracted from single‐cell datasets was used as the “signature matrix,” while the TCGA‐BLCA dataset and three GEO datasets were employed as reference matrices. Immune infiltration in TCGA‐BLCA samples was assessed using the CIBERSORTx LM22 signature matrix, which includes 22 validated leukocyte gene signatures.^[^ ^81^ ^]^ Quantile normalization was disabled in accordance with the recommendations provided by the CIBERSORTx web interface, and the number of permutations was set to 500.

### Cluster Analysis and Signature Scoring in TCGA‐BLCA Dataset

Based on the MMP11^+^mCAF‐related signature, consensus clustering analysis was performed on the TCGA‐BLCA dataset using ConsensusClusterPlus (v1.64.0).^[^ ^82^ ^]^ Consensus clustering was carried out using a hierarchical clustering algorithm with 100 iterations and a resampling rate of 80%. Tumor stromal score, immune score, tumor purity, and the ESTIMATE score were calculated using the ESTIMATE algorithm (v1.0.13). Additionally, the C3 signature score in TCGA‐BLCA samples was assessed using GSVA (v1.30.0) based on the MMP11^+^mCAF‐related signature.

### Immunohistochemical/Immunofluorescence Staining

For IHC staining, formalin‐fixed and paraffin‐embedded tissue samples were sectioned at a thickness of 5 µm. Following deparaffinization and rehydration, antigen retrieval was performed by heating the sections in 10 mm sodium citrate buffer (pH 6) at 100 °C for 10 min. Endogenous peroxidase activity was blocked using 0.3% hydrogen peroxide for 10 min. The sections were then incubated overnight at 4 °C with the primary antibodies. The following day, sections were treated with horseradish peroxidase‐conjugated secondary antibodies at room temperature for 30 min, and signal detection was achieved using 3,3′‐diaminobenzidine (DAB) as a chromogen. The sections were counterstained with hematoxylin, dehydrated, and imaged. The positively stained area and the number of microvessels at 20× magnification was quantified using ImageJ software (v1.53e, U.S. National Institutes of Health). Color deconvolution was applied to separate the DAB signal from the background. The “Analyze → Measure” function was used for quantification. For each sample, 3–5 randomly selected fields were analyzed, and the average value was used for statistical comparison.

For IF staining, antigen retrieval was performed as described above, followed by blocking with PBS containing 1% normal goat serum at room temperature for 1 h. Primary antibodies were applied to the sections and incubated overnight at 4 °C. After washing with PBS, Alexa Fluor 488‐ or Alexa Fluor 594‐conjugated secondary antibodies were added and incubated at room temperature for 1 h. Nuclei were counterstained with DAPI (Servicebio, China; GDP1024), and the sections were mounted using an anti‐fade mounting medium (Servicebio, China). Fluorescent images were captured using a Nikon Eclipse Ti‐SR fluorescence microscope (Nikon, Japan), and images were processed using CaseViewer software. The antibodies utilized in this study include anti‐COL1A1 (Servicebio, China; GB115707), anti‐MMP11 (Affinity, Jiangsu, China; AF0211), anti‐CD34 (Servicebio, China; GB15013), anti‐CD31 (Servicebio, China; GB11063), anti‐SPP1 (Servicebio, China; GB11500), and anti‐F4/80 (Servicebio, China; GB113373).

### Transwell Migration Assay

To evaluate cell migration, 500 µL of complete 10% fetal bovine serum (FBS) DMEM medium containing different treatments was added to the lower chamber of an 8 µm pore size Transwell insert (Corning Transwell, 24‐well plate). The treatments included 10 ng mL^−1^ WNT5A (Yeasen, Shanghai, China; 92282ES10), 10 µm Box5‐TFA (MedChemExpress, New Jersey, USA; HY‐123071A), a combination of the two agents, and PBS as a control. In the upper chamber, 100 µL of serum‐free DMEM containing 50 000 HUVEC cells was seeded. The cells were incubated at 37 °C for 6 h. After incubation, the Transwell inserts were removed, and the medium was carefully aspirated. Non‐migrated cells on the upper surface of the membrane were gently wiped off using a cotton swab. The inserts were then fixed with 4% paraformaldehyde for 20–30 min. After fixation, the inserts were rinsed with PBS. Migrated cells on the lower side of the membrane were stained with 0.1% crystal violet for 5–10 min, followed by three PBS washes to remove unbound dye. After air‐drying, the stained cells were observed and imaged using a light microscope Axio observer3 (Carl Zeiss, AG, Oberkochen, Germany) at 10× magnification, selecting 3–5 random fields per sample. Cell quantification was subsequently performed using ImageJ software (v1.53e, U.S. National Institutes of Health). For statistical analysis, each experimental group included three independent replicates, with at least three fields analyzed per replicate. The significance of differences between groups was determined using a Student's *t*‐test.

### Angiogenesis Assay

A mixture of Matrigel (Corning, NY, USA; 354230) and serum ‐ free DMEM (Gibco; 31885) was prepared at a 1:1 ratio. A total of 50 µL of this mixture was added to each well of a 96‐well cell culture plate and allowed to solidify at 37 °C for 3 h. HUVEC cells at passage five were seeded into the wells in a volume of 100 µL of medium, containing 50 000 cells per well. The cells were treated with different agents: 10 ng mL^−1^ WNT5A (Yeasen, Shanghai, China; 92282ES10), 10 µm Box5‐TFA (MedChemExpress, New Jersey, USA; HY‐123071A) and a combination of the two agents. Tube formation was monitored hourly under a light microscope Axio observer (Carl Zeiss, AG, Oberkochen, Germany), and representative images were captured. Tube formation metrics were quantified with ImageJ software (v1.53e, U.S. National Institutes of Health) using the Angiogenesis Analyzer plug‐in (http://image.bio.methods.free.fr/ImageJ/?Angiogenesis‐Analyzer‐for‐ImageJ). Statistical analysis was performed with three independent biological replicates per experimental group, each including at least three fields of analysis. Differences between groups were assessed using a Student's *t*‐test.

### Supernatant Transfer Experiments

Fibroblasts (HBdSF) were cultured until passage 3 (P3). The cells were then treated with a medium consisting of PBS, 5 ng mL^−1^ BMP2 (MedChemExpress, New Jersey, USA; HY‐P7006B), 10 ug mL^−1^ dorsomorphin (MedChemExpress, New Jersey, USA; HY‐13418A) or LDN193189 (MedChemExpress, New Jersey, USA; HY‐12071)and a combination of BMP2 and the inhibitor in DMEM for 24 h. Then, the supernatant was collected and mixed with DMEM supplemented with 10% FBS in a 1:1 ratio. For the migration assay, the supernatant mixture was added to the lower chamber of a Transwell system, and the remaining procedures were carried out according to standard Transwell migration protocols. Alternatively, for the angiogenesis assay, the supernatant was mixed with DMEM at a 1:1 ratio and used to resuspend HUVECs for subsequent experimentation. The cell quantification and tube formation metrics were calculated using the same methods as those employed in the Transwell migration and angiogenesis assays.

### Animal Model Experiment

All mice used in this study were maintained under specific pathogen‐free (SPF) conditions, and all experimental protocols were approved by the Institutional Ethics Committee of Shenzhen Luohu District People's Hospital. For tumor establishment, 20 µL of PBS containing 1 × 10^6^ MB49 tumor cells was injected into the bladder of 6–8‐week‐old C57BL/6 mice. One week after tumor cell injection, the tumor‐bearing mice were randomly divided into four groups (*n* = 6 per group) and received intraperitoneal injections every two days with PBS, 2 µg kg^−1^ BMP2 (MedChemExpress, New Jersey, USA; HY‐P7006B), 5 mg kg^−1^ Dorsomorphin (MedChemExpress, New Jersey, USA; HY‐13418A), or a combination of the two agents. Tumor growth was monitored and recorded using a multimodal in vivo imaging system (AniView100, BioLight, Guangzhou, China) throughout the experiment. After 27 days of treatment, tumors were harvested and weighed. Portions of the tumor tissues were processed for paraffin embedding, sectioning, IHC staining, and flow cytometry analysis.

### Quantitative Real‐Time PCR (qRT‐PCR) Analysis

HUVEC cells were treated with PBS, WNT5A (10 ng mL^−1^), WNT5A + Box5‐TFA (10 µm), or Box5‐TFA (10 µm) for 12 h. HBdSF fibroblast cells were treated with PBS, BMP2 (5 ng mL^−1^), LDN193189 (10 ug mL^−1^, MedChemExpress, New Jersey, USA; HY‐12071), or BMP2 + LDN193189 for 12 h. Total RNA was extracted using TRIzol reagent (Invitrogen, 15596026CN) according to the manufacturer's instructions. The RNA concentration and purity were assessed using a NanoDrop spectrophotometer (Thermo Fisher Scientific, USA). Reverse transcription was performed using the HiScript III All‐in‐One RT SuperMix Perfect for qPCR (Vazyme, China; R333‐01) following the manufacturer's protocol. qRT‐PCR was conducted using the ChamQ SYBR qPCR Master Mix (Vazyme, China; Q311) on a QuantStudio Dx Real‐Time PCR System (Applied Biosystems, USA). The reaction conditions were as follows: 95 °C for 30 s, followed by 40 cycles of 95 °C for 10 s and 60 °C for 30 s. Gene‐specific primers used for qRT‐PCR are listed in Table S4 (Supporting Information). The β‐actin (*ACTB*) gene was used as an internal control for normalization. Relative gene expression levels were calculated using the 2^‐ΔΔCt^ method. Each experiment was performed in triplicate, and data were presented as mean ± standard deviation (SD).

### Flow Cytometry

Tumor samples were minced and digested in DMEM (Gibco) containing collagenase I (1 mg mL^−1^, Sigma), collagenase IV (1 mg mL^−1^, Sigma), DNase I (5 mg mL^−1^, Roche), and 2% FBS (Gibco) at 37 °C for 1 h. The resulting cell suspension was filtered through a 40 µm cell strainer and treated with red blood cell lysis buffer (Gibco) for 3 min. Cells were then resuspended in FACS buffer and incubated with fluorochrome‐conjugated antibodies FITC Plus Anti‐Mouse CD31 (Proteintech, FITC‐65058) at 4 °C in the dark for 40 min. After two washes with cold PBS, the samples were analyzed using a BD FACS Canto II flow cytometer (BD Bioscience). Data were processed and analyzed using FlowJo software (FlowJo, LLC, Ashland, Oregon).

### Statistical Analysis

All statistical analyses and visualizations were performed using R (v4.0.3 and v4.3.3), Python (v3.7.0), and GraphPad Prism 8. Survival data were analyzed using the Kaplan‐Meier method and the log‐rank test. Optimal cut‐off values were determined to stratify cell proportions or gene set expression levels for survival analysis. Spearman's correlation coefficient was used to evaluate linear relationships. *p*‐values indicated in the figures denote statistical significance, with *p* < 0.05 considered statistically significant.

## Conflict of Interest

The authors declare no conflict of interest.

## Author Contributions

W.X. and T.L. contributed equally to this work (co‐first authors). W.X. and T.L. contributed to the design of the study, analyzed data, prepared figures, and edited the manuscript with help from H.F. D.D., X.T., and L.F. performed or contributed to all experiments, with help from D.T., L.L., and L.F. performed cell experiments. D.D., X.T., Q.D., H.Z., and X.H. performed mouse experiments and flow cytometry assay. D.T. provided the paraffin‐embedded samples. F.L. collected the public datasets. S.W., T.T., and D.F. supervised the study. All authors read and edited the manuscript.

## Code Availability Statement

R and Python scripts used to analyze data and generate figures are available upon request to the corresponding author.

## Supporting information



Supporting Information

## Data Availability

The data that support the findings of this study are available from the corresponding author upon reasonable request.
