# Supplementary material for: Single‐Cell RNA Sequencing Identifies MMP11+ Cancer‐Associated Fibroblasts as Drivers of Angiogenesis and Bladder Cancer Progression
Source: Adv Sci (Weinh). 2025 Jun 24;12(32):e02774. doi: 10.1002/advs.202502774 (PMC12407355; doi:10.1002/advs.202502774)
Supplement: Supplementary file 1 — Supporting Information [file ADVS-12-e02774-s001.pdf]

ADVANCED SCIENCE Open Access

Supporting Information

for *Adv. Sci.*, DOI 10.1002/advs.202502774

Single-Cell RNA Sequencing Identifies MMP11^{+} Cancer-Associated Fibroblasts as Drivers of Angiogenesis and Bladder Cancer Progression

Wuwu Xu, Ting Liang, Hu Fang, Lu Fu, Dashi Deng, Xiyang Tan, Lisha Liu, Dongdong Tang, Haoxiang Zheng, Qiuxia Ding, Xiuqi Hou, Daquan Feng, Tao Tao and Song Wu**

Supporting Information

Single-Cell RNA Sequencing Identifies MMP11$^+$ Cancer-Associated Fibroblasts as Drivers of Angiogenesis and Bladder Cancer Progression

Wuwu Xu[1,2#], Ting Liang[5,1#], Hu Fang[3], Lu Fu[1,2], Dashi Deng[1,2], Xiyang Tan[1,2], Lisha Liu[1,2], Dongdong Tang[4], Haoxiang Zheng[3], Qiuxia Ding[1,2], Xiuqi Hou[1], Daquan Feng[5], Tao Tao[1,2*] and Song Wu[1,2,3*]

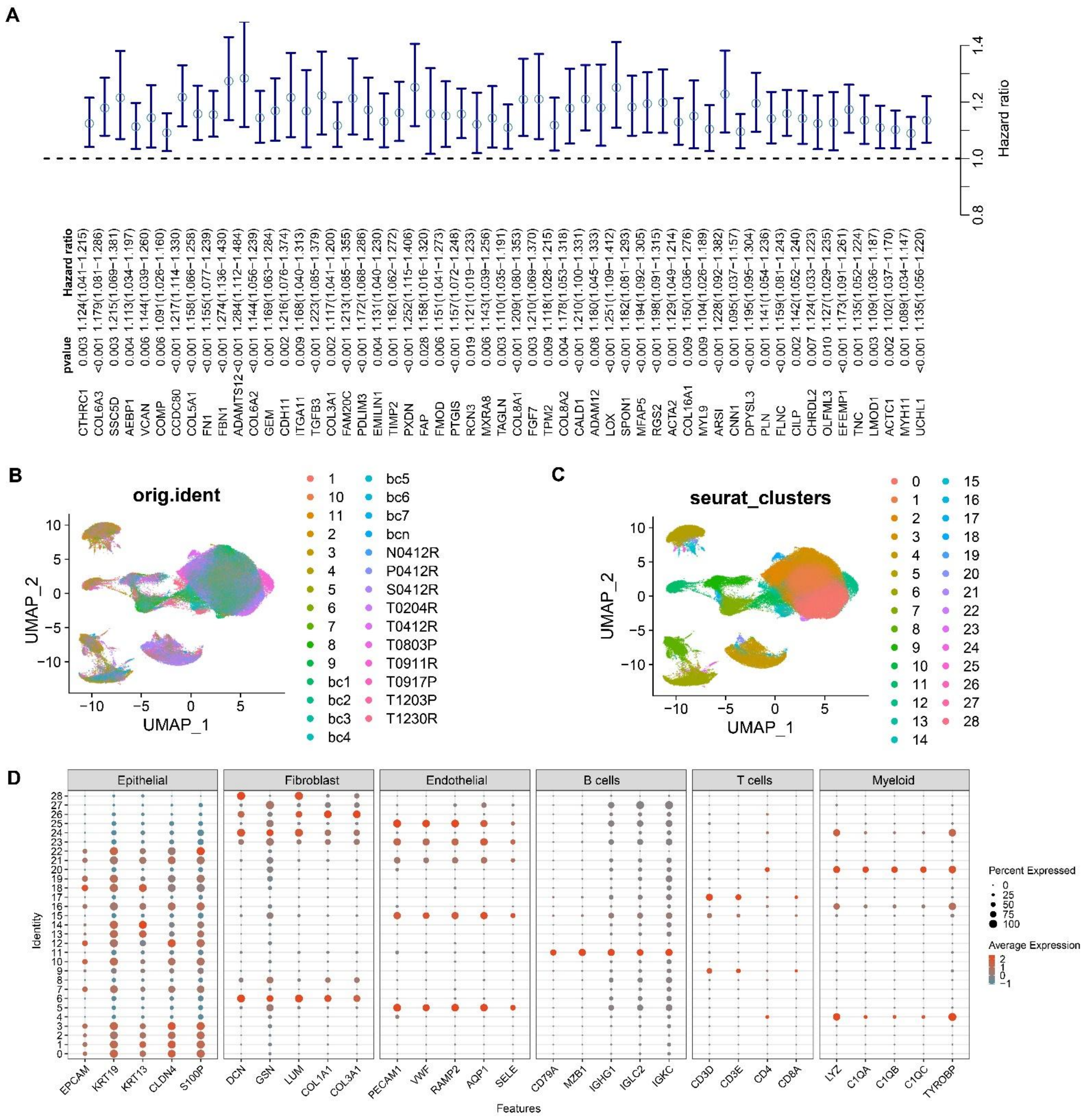

Fig.S1 Summary of HR gene analysis and single-cell clustering results.

A, Univariate Cox regression analysis of HR genes. B-C, UMAP plots showing the sample origin of all cells (B) and Seurat-based clustering (C). D, DotPlot illustrating the expression of marker genes across different cell types in Seurat clusters.

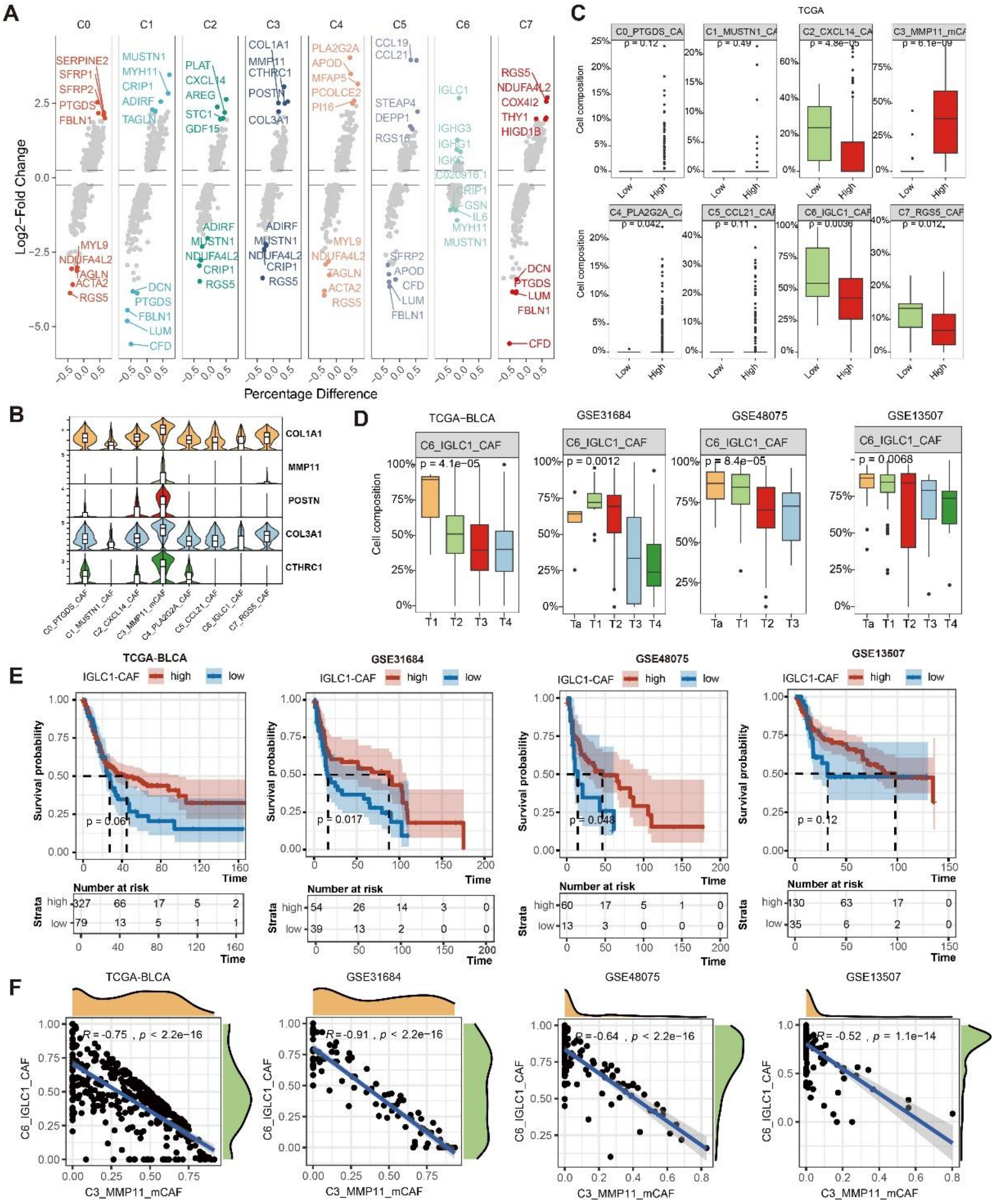

Fig.S2 Fibroblast subcluster analysis and clinical relevance.

A, Differentially expressed genes (upregulated and downregulated) across fibroblast subtypes. B, Violin plot showing the expression of characteristic genes in MMP11^{+} mCAFs. C, CIBERSORT deconvolution analysis of fibroblast subclusters in the TCGA-BLCA dataset, comparing their proportions in patients with high-grade versus low-grade tumors. Statistical analysis was performed using the Wilcoxon test. D, Proportion of IGLC1^{+} CAFs in each clinical stage across four datasets, as assessed by CIBERSORT deconvolution analysis. Statistical analysis was performed using the Kruskal test. E, Kaplan–Meier survival curves depicting the overall survival of patients grouped by high and low proportions of IGLC1^{+} CAFs across four bladder cancer cohorts. The log-rank test was used to assess statistical significance. "High" and "Low" indicate patient groups with relatively high or low levels of IGLC1^{+} CAFs. F, Spearman correlation analysis showing a significant negative correlation between the proportions of IGLC1^{+} CAFs and MMP11^{+} mCAFs across four bladder cancer cohorts.

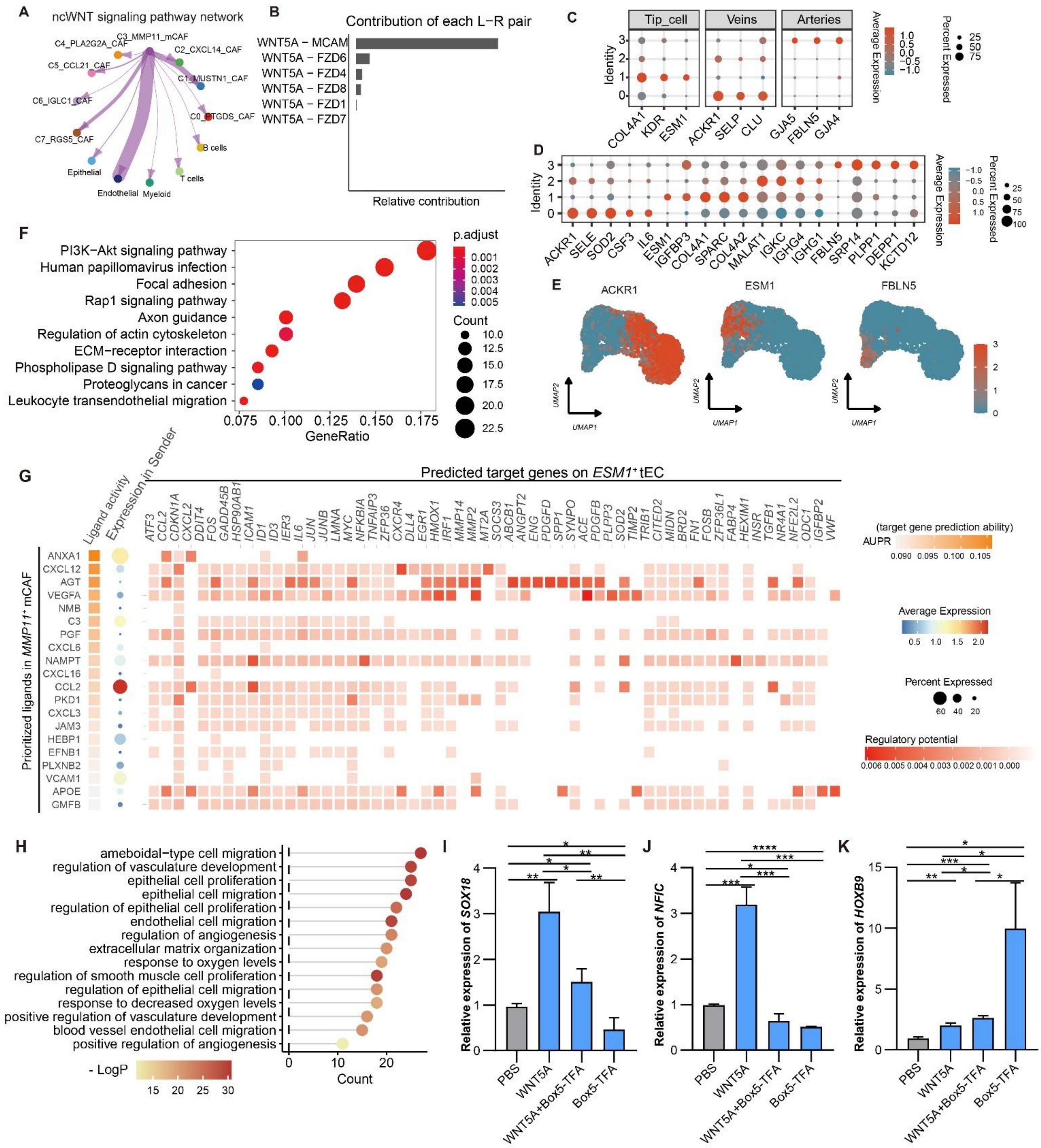

Fig. S3 A, Communication strength of the ncWNT signaling pathway network between MMP11⁺ mCAFs and other cell populations. B, Relative contribution of ligand-receptor pairs within the ncWNT signaling pathway. C, Expression of venous, arterial, and tip cell marker genes in endothelial cell Seurat clusters. D, Differentially expressed genes across endothelial cell clusters. E, UMAP plots showing the expression of endothelial marker genes. F, KEGG enrichment analysis of upregulated genes in ESM1⁺ tECs. G, NechNet analysis displaying intercellular communication between MMP11⁺ mCAFs and SPP1⁺ macrophages, showing ligand activity, gene expression, and ligand-regulated target genes. H, GO enrichment analysis of target genes regulated by MMP11⁺ mCAFs in SPP1⁺ macrophages. I-K, Relative expression of *SOX18* (I), *NFIC* (J) and *HOXB9*. Data are presented as mean ± standard deviation (SD) from at least three independent experiments. Statistical significance was determined using Student's *t*-test; *$p < 0.05$; **$p < 0.01$; ***$p < 0.001$; ****$p < 0.0001$.

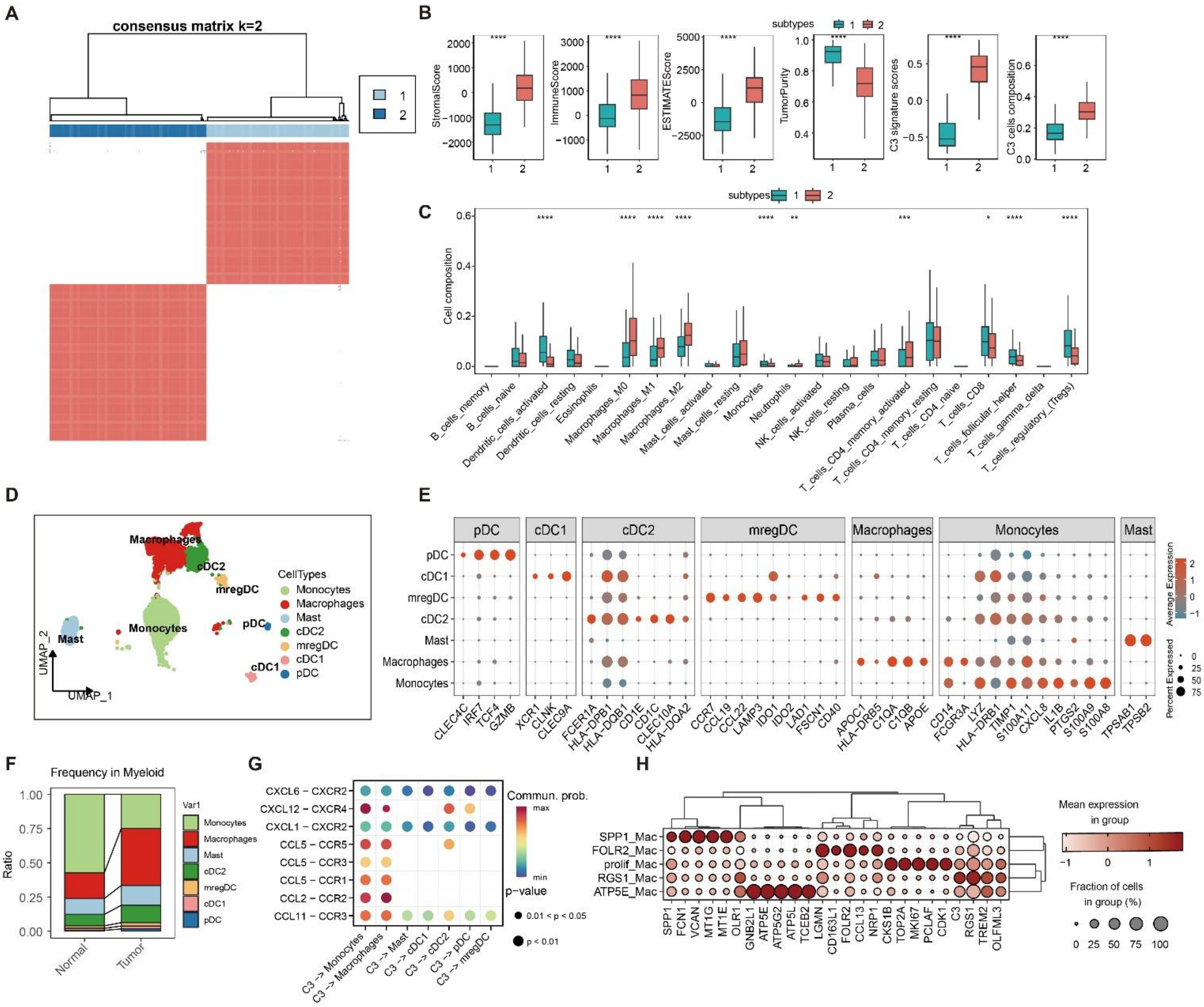

Fig.S4 Immune infiltration patterns of MMP11^{+} mCAF-related subtypes and classification of myeloid cells.

A, TCGA-BLCA cohort stratified into two molecular subtypes based on the MMP11^{+} mCAF signature. B, Comparison of stromal score, immune score, ESTIMATE score, tumor purity, MMP11^{+} mCAF signature score, and MMP11^{+} mCAF cell composition between the two subtypes. C, Infiltration abundances of 22 immune cell types between the two subtypes. D, UMAP plot illustrating the classification of myeloid cell populations. E, Expression of marker genes for myeloid cell subpopulations. F, Proportional distribution of different myeloid cell types in tumor versus normal tissues based on single-cell datasets. G, Cell-cell communication between MMP11^{+} mCAFs and myeloid cell populations. H, DotPlot showing the expression of differentially expressed genes among macrophage subclusters.

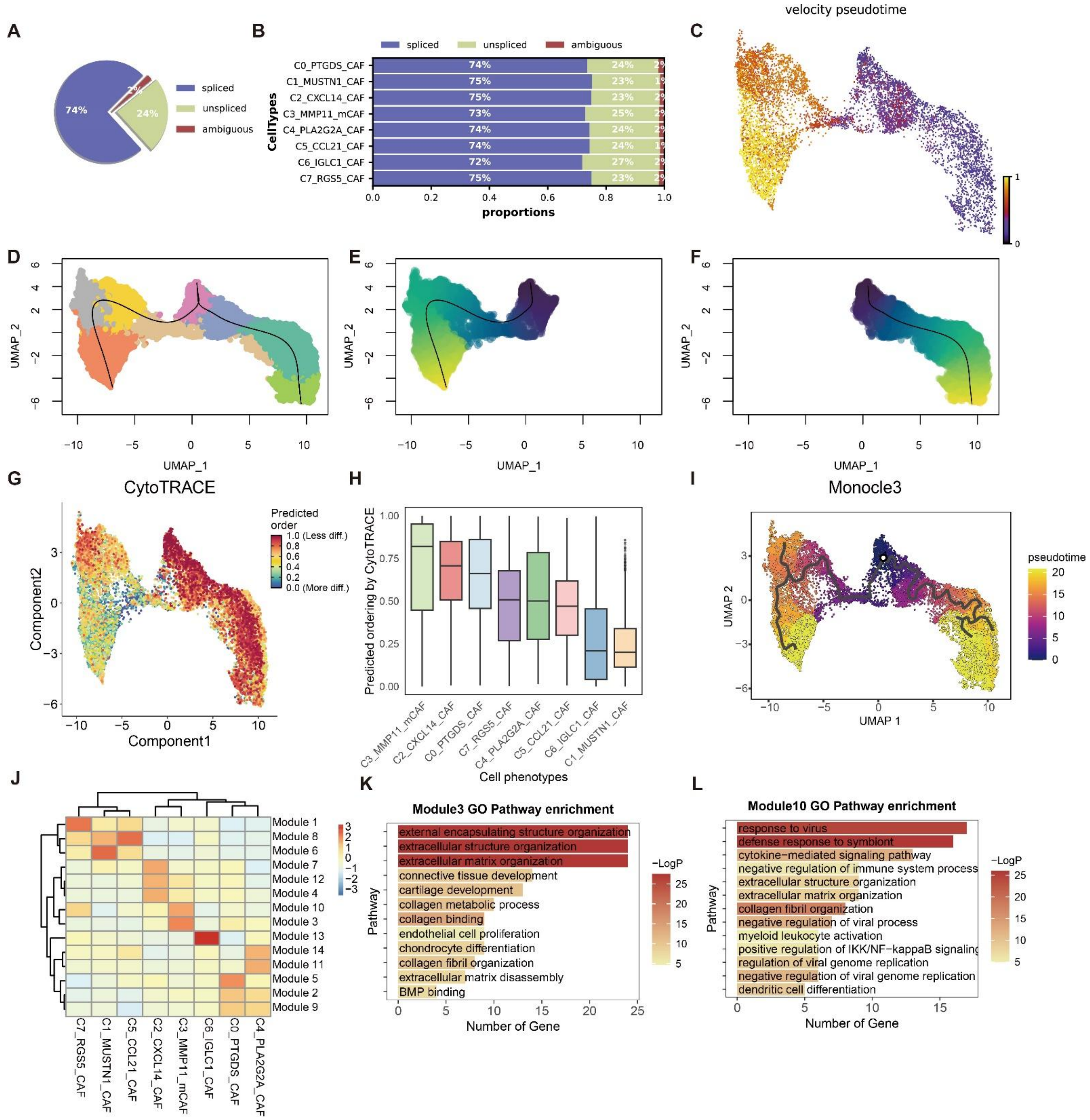

Fig. S5 Analysis of fibroblast differentiation trajectories.

A, Pie chart illustrating the overall proportion of spliced and unspliced RNA. B, Stacked bar plot showing the distribution of spliced and unspliced RNA across different fibroblast subpopulations. C, UMAP projection depicting RNA velocity-based inferred pseudotime of fibroblasts. D, UMAP plot displaying two predicted developmental trajectories derived from Slingshot analysis, with cells color-coded by cell type. E-F, UMAP plots showing the predicted developmental trajectories 1 (E) and 2 (F) inferred using Slingshot analysis, with cells color-coded by pseudotime predictions. G, UMAP plots visualizing the distribution of CytoTRACE scores across fibroblasts. "Less diff." indicates less differentiated states, while "More diff." indicates more differentiated states. H, Box plot illustrating CytoTRACE scores for all fibroblast subpopulations, arranged along the x-axis from left to right in order of increasing differentiation status. I, Visualization of fibroblast developmental trajectories inferred using Monocle3. J, Pseudotime-associated co-expression gene modules identified within fibroblast subpopulations. K-L, GO enrichment analysis of co-expression gene module 3 (K) and module 10 (L).

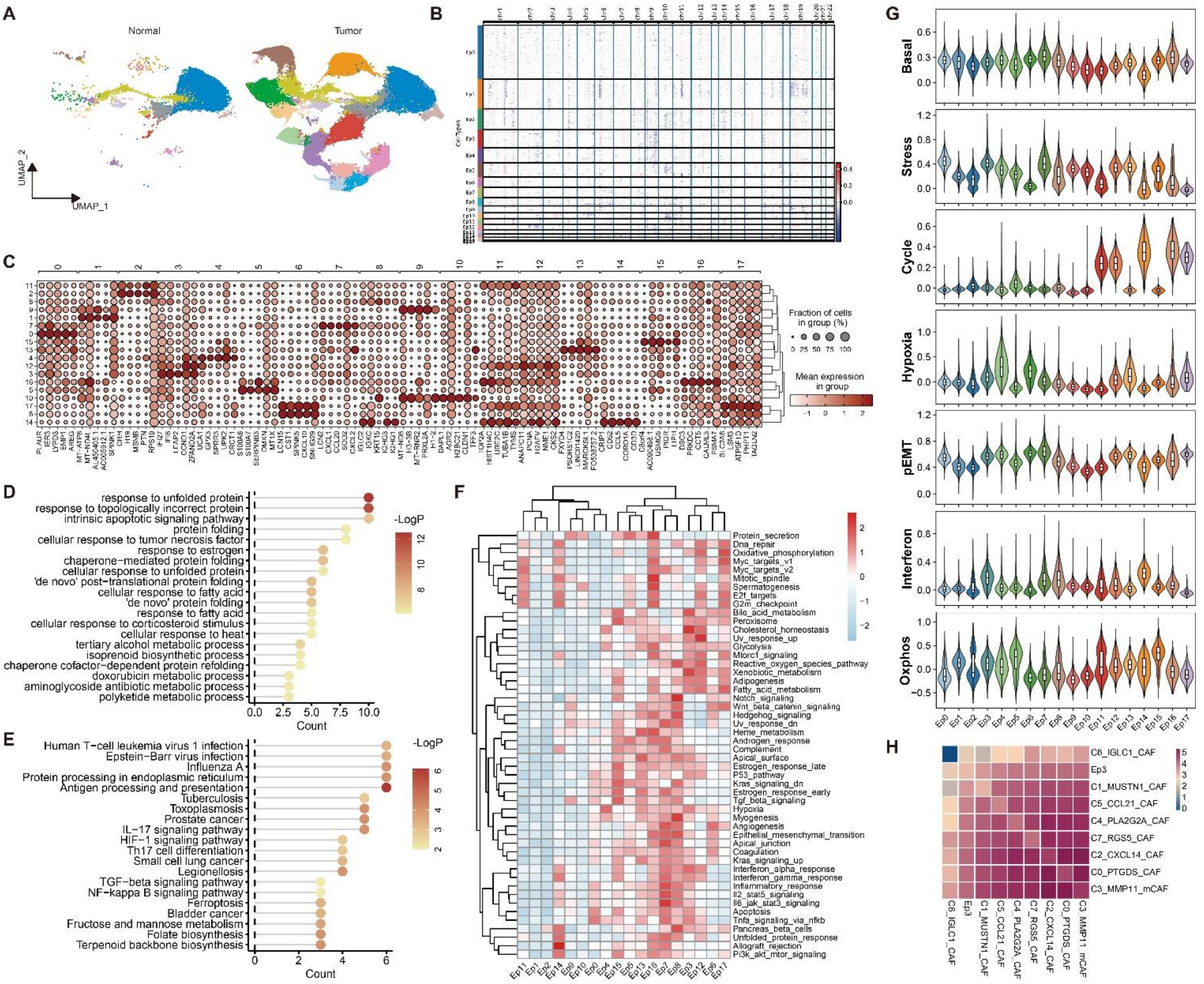

Fig.S6 Identification and characterization of epithelial cells.

A, UMAP plot showing the distribution of epithelial cell populations in normal and tumor tissues. B, Copy number variation (CNV) analysis across all epithelial cells, with epithelial cells from normal tissue as the baseline reference. Red indicates amplifications, blue represents deletions, and columns are sorted by chromosomal position. C, DotPlot displaying differentially expressed genes among epithelial subpopulations. D-E, GO (D) and KEGG (E) enrichment analyses of upregulated genes in the Ep3 cell population. F, Heatmap showing variations in pathway activities, scored by GSVA, across all epithelial groups. G, Gene set scoring analysis for cell states across epithelial subpopulations, including Basal, Stress, Hypoxia, pEMT, Interferon, and Oxphos states. H, Heatmap illustrating the interaction strength between Ep3 cells and fibroblast subpopulations.

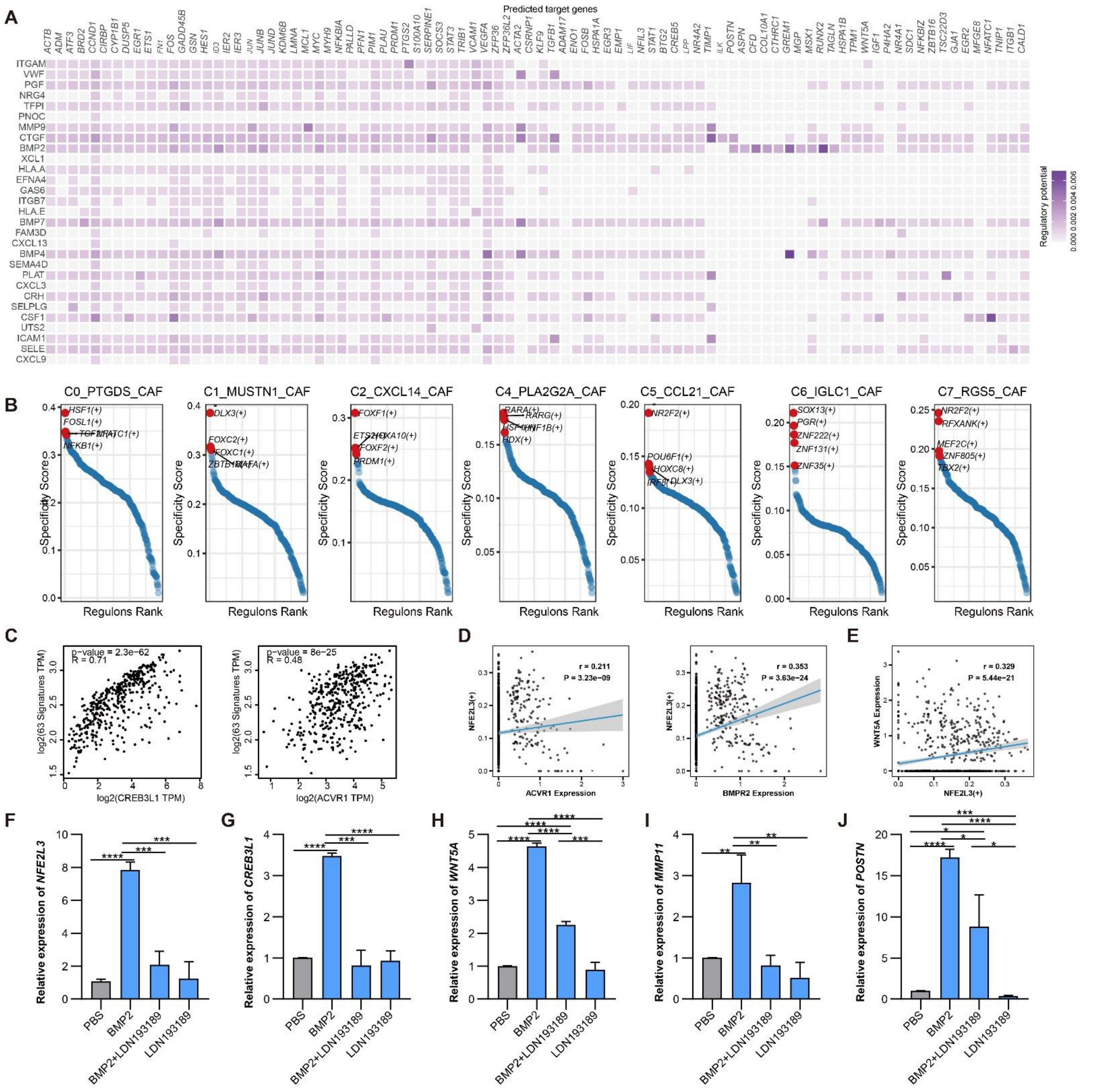

Fig.S7 Transcription Factor Analysis of Fibroblast Subpopulations

A, A heatmap displaying the potential regulatory ligands and their target genes for the MMP11[+] mCAF cell subset in the TME, derived using NechNet analysis. B, Specific transcription factors of fibroblast subpopulations. C, Spearman correlation analysis showing a significant positive correlation between the expression of the MMP11[+] mCAF signature gene set and the transcription factors *CREB3L1* and *ACVR1* in the TCGA-BLCA cohort. D, Spearman correlation analysis revealing a significant positive correlation between the activity of NFE2L3 and the expression of BMP signaling receptor genes in the MMP11[+] mCAF cell population. E, Spearman correlation analysis indicating a significant positive correlation between the expression of *WNT5A* and the activity of NFE2L3 in the MMP11[+] mCAF subset. F-J, Relative expression of *NFE2L3* (F), *CREB3L1* (G), *WNT5A* (H), *MMP11* (I) and *POSTN* (J). Data are presented as mean ± standard deviation (SD) from three independent experiments. Statistical significance was determined using Student's *t*-test; *$p < 0.05$; **$p < 0.01$; ***$p < 0.001$; ****$p < 0.0001$.

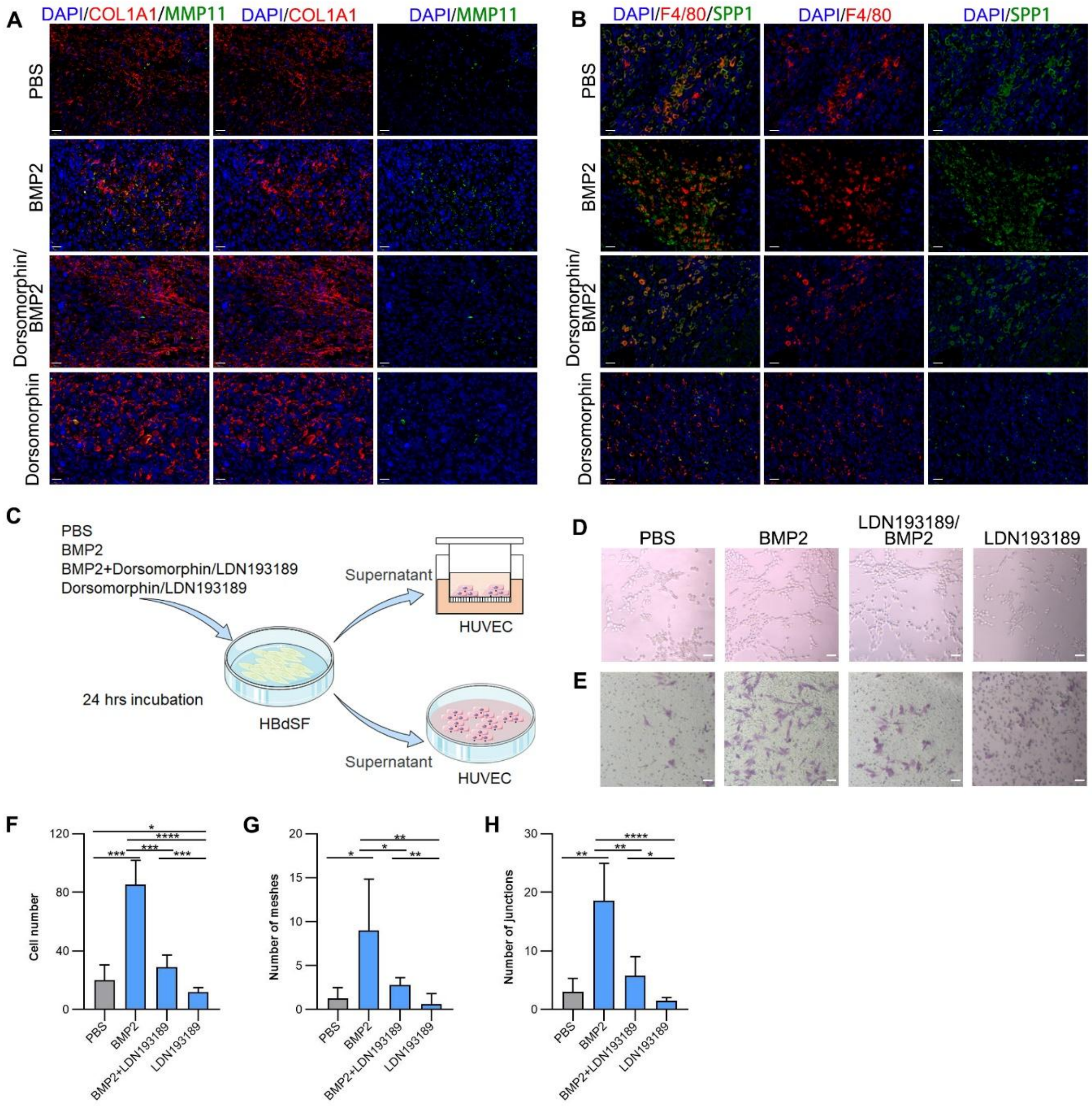

Fig.S8 A-B, IF analysis showing MMP11^{+} mCAF (A) and SPP1^{+} macrophage (B) in tumors from different treatment groups, scale bar=20 μm. C, Schematic diagram of the conditioned medium transfer experiment for assessing endothelial cell migration and tube formation capacity. D-E, Representative images of tube formation (D) and Transwell migration (E) assay of HUVEC treated with conditioned media derived from HBdSFs after treatment with BMP2 or LDN193189, scale bar=50 μm. F. Quantification of migrated HUVECs in the Transwell assay (E). G-H. Quantification of mesh formation (G) and junction number (H) in the tube formation assay (D). Data are presented as mean ± SD. Statistical analysis was performed using Student's *t*-test; *$p < 0.05$; **$p< 0.01$; ***$p< 0.001$; ****$p < 0.0001$.

Supplementary Table 1. Upregulated genes associated with poor prognosis and downregulated genes associated with favorable prognosis in stage III-IV tumor samples.

Genes	p.value	HR	Low 95%CI	High 95%CI	Log2FC
CTHRC1	0.0421	1.3663224	1.011205834	1.846149201	1.71158054836901
COL6A3	0.0328942	1.3873523	1.026969206	1.874200661	1.47821425716633
CCN2	0.0128254	1.4646544	1.084438634	1.978177888	1.43924353309293
SSC5D	0.0351847	1.3836528	1.02280621	1.871806245	1.07410607336499
AEBP1	0.0441021	1.3629486	1.008209583	1.842502779	1.63046104162903
VCAN	0.0144024	1.4597893	1.07820894	1.976411841	1.37322456148336
COMP	0.0063891	1.5247149	1.125983238	2.064644792	2.03036649509331
CCDC80	0.0013626	1.6448501	1.212977158	2.230488794	1.39523289399905
COL5A1	0.0232059	1.4187475	1.048935465	1.918940164	1.45362820922465
FN1	0.0084473	1.5004581	1.109373508	2.029410737	1.77844519354024
FBN1	0.034547	1.3821811	1.023809929	1.865995317	1.16344862589721
ADAMTS12	0.0053979	1.5383726	1.135760719	2.083705042	1.01778823716658
COL6A2	0.0185522	1.4339316	1.062243083	1.935677266	1.40058918083177
GEM	0.0186124	1.4338789	1.062073489	1.935844198	1.18529596346787
CDH11	0.0062575	1.5282833	1.127585661	2.071372356	1.07349422696801
ITGA11	0.0058001	1.5354489	1.13221674	2.082289684	1.11408337245216
TGFB3	0.0097457	1.4849614	1.100286402	2.00412396	1.00915880269792
COL3A1	0.0378964	1.3775864	1.018062032	1.864075409	1.56208380958043
FAM20C	0.0244737	1.4113461	1.045359982	1.905465974	1.1639643831526
PDLIM3	0.0015927	1.6331751	1.204439978	2.214523628	1.14744297851974
EMILIN1	0.0180143	1.4405342	1.064552575	1.949306092	1.30156214884208
TIMP2	0.0188077	1.4334843	1.061498171	1.935827484	1.21368189889709
PXDN	0.0241095	1.4135598	1.046377835	1.90958859	1.04892069930322
FAP	0.0355949	1.3797991	1.021922965	1.86300309	1.05441885772736
FMOD	0.0013435	1.6412329	1.212409124	2.221729773	1.15314075003639
PTGIS	0.0006796	1.6956695	1.250389686	2.299519156	1.46196216559181
INHBA	0.0202069	1.4271688	1.057090863	1.926807779	1.24097277239393
RCN3	0.0242907	1.4144259	1.046058777	1.912512607	1.12069310651779
MXRA8	0.0340386	1.3863901	1.024912603	1.875357593	1.09946702322393
TAGLN	0.0048573	1.5454232	1.141523053	2.092233635	1.3205088081199
COL8A1	0.0222365	1.4247759	1.051829401	1.929957919	1.09344365219415
FGF7	0.0215383	1.4247605	1.053510707	1.926836164	1.01531367270101
TPM2	0.012068	1.4705055	1.088193511	1.987134014	1.1455017485688
COL8A2	0.0035752	1.571899	1.159528639	2.130923319	1.04743772252981
CALD1	0.0037666	1.5675615	1.156508441	2.124713464	1.02311359030059
ADAM12	0.0142485	1.4618222	1.079025652	1.980420148	1.05249977943882
LOX	0.044907	1.3610254	1.00702705	1.839464196	1.01019132108454
SPON1	0.001041	1.6632878	1.227147132	2.25443724	1.15385079385877
MFAP5	0.0374506	1.3771043	1.018758423	1.861497567	1.2829491806376
RGS2	0.0047659	1.544668	1.142099747	2.089133821	1.03603272352098
ACTA2	0.0065725	1.5237989	1.124620789	2.064663144	1.20434341944492
COL16A1	0.0404434	1.3694871	1.013785629	1.849991697	1.08421531105107
MYL9	0.0286191	1.3976794	1.035609118	1.886336937	1.14068380151897
ARSI	0.0342406	1.3841242	1.024439126	1.870096259	1.07721588974934
FSTL3	0.0464519	1.3553671	1.004791011	1.828260837	1.0426497728458
CNN1	0.0265578	1.4059198	1.040425048	1.899810606	1.57798366475357
THBS2	0.0106706	1.4856303	1.096349288	2.013133545	1.29898971948419
DPYSL3	0.0257766	1.4066009	1.042117529	1.898563169	1.11627014901373
PLN	0.0063925	1.5233099	1.125668971	2.061416824	1.11831102912406
FLNC	0.0003551	1.745369	1.285701802	2.36937763	1.18000607224213
CILP	0.017624	1.4381218	1.065341051	1.941344769	1.15063590231602
CHRDL2	0.0042528	1.5516584	1.148119479	2.097032533	1.22576706609552

OLFML3	0.0174397	1.4424303	1.066422649	1.951013541	1.03925884748106
EFEMP1	0.0001202	1.8217603	1.341936743	2.473149923	1.33010822796174
TNC	0.0109734	1.4740435	1.093106267	1.987733868	1.21481114047234
LMOD1	0.0159837	1.4461031	1.071214723	1.952189445	1.05280384703319
ACTC1	0.004098	1.5559921	1.150552511	2.104303313	1.26980837749368
MYH11	0.0044785	1.5478266	1.145240803	2.09193326	1.17576006529758
UCHL1	0.0004578	1.717671	1.269223239	2.324566319	1.01664937021256
CTSE	0.0057592	0.6548915	0.484932589	0.884417572	-1.32253343
CRTAC1	0.0106304	0.6776421	0.502735881	0.913399778	-1.193683354
SLC44A4	0.0209429	0.7035524	0.522007557	0.948235313	-1.128786716
HMGCS2	0.027308	0.7126615	0.527520035	0.962781353	-1.472850987
CYP4B1	0.0300166	0.7172333	0.531231611	0.968360284	-1.182618299
DHRS2	0.0083385	0.6655445	0.491818448	0.900636161	-1.273000011
SPINK1	0.0461754	0.7374385	0.546632532	0.994846545	-1.190745299

Supplementary Table 2. Clinical information of patients in house corhort

Idents	Gender	Age	Grade	Invasiveness	Tumor Status	Tumorstage	Tissue
N0412R	M	59	-	-	-	-	Normal
T0412R	M	59	High	Invasive	Recurrence	T2	Tumor
T1203P	F	78	High	Invasive	Primary	T1	Tumor
P0412R	M	59	-	-	-	-	Normal
T0911R	M	58	High	Invasive	Recurrence	T2	Tumor
S0412R	M	59	-	-	-	-	Normal
T0204R	M	59	High	Invasive	Recurrence	T2	Tumor
T0803P	M	58	High	Invasive	Primary	T2	Tumor
T0917P	M	28	Low	Noninvasive	Primary	T0	Tumor
T1230R	M	61	High	Invasive	Recurrence	T3	Tumor

Supplementary Table 3. Signature of fibroblast clusters

**C0_PTGDS _CAF**	**C1_MUSTN1 _CAF**	**C2_CXCL14 _CAF**	**C3_MMP11_ mCAF**	**C4_PLA2G2 A_CAF**	**C5_CCL21 _CAF**	**C6_IGLC1 _CAF**	**C7_RGS5_ CAF**
PTGDS	MUSTN1	CXCL14	COL11A1	MFAP5	CCL19	IGKC	NDUFA4L 2
SERPINE2	MYH11	PLAT	COL10A1	PI16	CCL21	IGHG1	RGS5
FBLN1	CRIP1	STC1	POSTN	PCOLCE2	STEAP4	IGLC1	COX4I2
SFRP2	ADIRF	SLC14A1	ARL4C	EFEMP1	CFHR1	KRT17	THY1
SFRP1	TAGLN	NRG1	COL1A1	C3	ARHGAP15	C1orf56	HIGD1B
CFD	PLN	HSD17B2	DIO2	CFD	HOPX	HEXIM1	COL4A1
TNFAIP6	MYL9	TMEM176B	NTM	GSN	SSTR2	CD74	IFI27
CYR61	BCAM	BMP5	MFAP2	CCDC80	SYNPO2	VMP1	ARHGDIB
IGF1	HES4	TRPA1	MMP11	SFRP4	PTGER1	HLA-DRA	PPP1R14A
APOE	PTP4A3	CYGB	COL1A2	ITM2A	FHL5	MALAT1	COL4A2
GSN	SORBS2	PITX1	COL3A1	OGN	LGI4	KRT19	CYTOR
MATN2	ADRA2A	PDGFRA	CTHRC1	DCN	TINAGL1	IGHG3	FRZB
GNB2L1	DES	WNT5A	COL5A1	CILP	NR2F2	CD24	EGFL6
ITM2A	DSTN	FENDRR	SFRP4	FBLN2	LHFPL6	AC020916.1	ANGPT2
PTGS2	TPM2	PDGFD	COL12A1	CYP1B1	RGS16		COL18A1
RGCC	SPARCL1	ENPP2	SPARC	CD34	ECRG4		GJA4

LMCD1	ACTA2	NDNF	COL8A1	OMD	SLC7A2		TPPP3
DCN	LBH	OTULINL	MXRA5	VIT	IFITM2		CHN1
TFPI2	ID4	EMID1	COL6A3	CLU	IGFBP7		CCDC102B
FBLN2	GADD45B	BMP4	VCAN	PLA2G2A	NOTCH3		NOTCH3
SEPP1	RERGL	COL8A1	THBS2	SEMA3C	MTHFD2		PLXDC1
MGP	MCAM	TMEM119	SULF1	ADH1B	DEPP1		FJX1
MMP2	PHLDA2	HLA-A	ADAM12	TSPAN8	ID4		ITGA1
CLU	CNN1	RARRES2	FAP	S100A10	CD44		MEF2C
DPT	RHOB	TGFBI	INHBA	APOD	IFITM1		MCAM
EMP1	RGS5	AKR1B1	MMP14	TNXB	CD59		IGFBP7
SERPINE1	SNCG	MFAP4	RARRES2	UAP1	GJA4		ESAM
CSF3	TINAGL1	LUM	LGALS1	PTGIS	C20orf27		CDH6
MGST1	FRZB	F3	PDPN	LINC01133	EPAS1		MYO1B
QSOX1	CSRP1	HLA-C	COL5A2	IGFBP6	CPE		FOXS1
CHRDL2	C11orf96	HHIP	LUM	SCARA5	EPS8		GUCY1A2
FST	APOLD1	ADM	ISLR	HAS1	MAP1B		TESC
BASP1	FLNA	ITM2C	NREP	CXCL14	CNN3		GMFG
LUM	CAVIN3	PDPN	TMEM158	SLPI	IGFBP2		GJC1
MMP23B	CRIP2	TMEM176A	NBL1	PDGFRL	SMOC2		ADGRF5
RHOBTB3	MAP3K7CL	GDF15	LOXL1	RAMP2	ADAMTS4		ADAP2
PTX3	DBNDD2	ATP1B1	CDH11	SFRP2	ACTA2		LURAP1L
PLTP	NRGN	EMILIN1	MMP2	MGST1	COL4A1		PDGFRB
THBS1	MYLK	ENC1	FN1	CST3	CPM		SEPTIN4
PTN	TPM1	ITGA8	RCN3	CYBRD1	MT1M		OLFML2A
SELM	MFGE8	LTBP4	MRC2	SERPINF1	MT1A		GUCY1B1
GLTSCR2	SYNM	NBL1	ANTXR1	FBLN5	FGF7		PDGFA
SERPINF1	PPP1R14A	TGM2	GREM1	FBLN1	FABP4		PTP4A3
GPRC5A	ACTG2	ABCA8	CERCAM	GPNMB	SOCS3		EHD2
SLC19A2	ZFHX3	SLC9A3R2	PLAU	GPC3	CXCL2		CALD1
NPC2	NTRK2	LGALS3BP	CCL11	C1R	CCL2		SPARC
OGN	SLC25A4	GJA1	CHI3L1	ANXA2			BGN
HTRA1	CSRP2	BST2	EMILIN1	FBN1			GAPDH
PI16	MYL6	TCIM	IL32	ABI3BP			LOXL2
COL14A1	NET1	C1S	FKBP10	CD55			TBX2
GPNMB	RCAN2	DKK3	COL6A1	MGP			PGF
C6orf48	HRH2	PLPP1	MXRA8	CLEC3B			CSPG4
LINC01082	NRARP	APCDD1	MARCKS	HTRA3			DBNDD2
SOD2	CCDC3	KRT17	CTSK	CTGF			B2M
MEG3	NOTCH3	AREG	COL15A1	S100A13			WFDC1
SMPDL3A	NDUFA4L2	TCF21	SPON2	MEDAG			FAM162B
CFH	GJA4	FHL2	SERPINH1	SERPING1			CRIP1
C7	EFHD1	AGT	MDK	PLAC9			MIR4435-2HG
ADH1B	SGCA	OSR2	AEBP1	CYR61			OAZ2
CCDC80	DMPK	IL32	BGN	S100A6			CRIP2
PTGES	ITGA7	TMEM158	IGHG4	IGF1			CFL1
SPON1	PRPH	CTSC	IGKC	ATP5E			KRT18
FTL	SELENOW	CCN1	IGLC2	ADD3			ISG15
SRPX	HEYL	NCOA7		CADM3			TINAGL1
PLPP3	NUDT4	PLAU		C1S			NR2F2
FBLN5	PGF	CCN2		FSTL1			RHOC
FGF7	HSPB1	IGFBP3		SLIT3			HOPX
AC090498.1	LMOD1	CXCL8		ANXA1			UACA

CREB5	ADAMTS9	IGFBP2		EMP1			PPIA
RCAN1	TSC22D1			DPT			PFN1
BDKRB1	PDGFA			TCEB2			HLA-B
TNXB	TBX2-AS1			ATP5I			F2R
LIF	TBC1D1			GNB2L1			SEPTIN11
EFEMP1	C1QTNF1			CFH			CLIC1
TGFBR3	CDKN1A			FGF7			MARCKSL1
FCGRT	ARPC1A			C14orf2			LHFPL6
ELN	CRYAB			SDCBP			PAG1
SERPINA3	MGST3			VIMP			CHCHD10
OLFML3	YBX3			ATP5L			NREP
ABI3BP	JAG1			PRELP			KCNJ8
CYBRD1	LPP			PRKCDBP			ID3
CXCL12	ADAMTS1			H19			MAP1B
MFAP4	CAVIN1			SEPW1			KCNE4
UGDH	CCDC107			PODN			SERPINI1
SAT1	PPP1R12B			GPX3			SEPTIN7
ABL2	AK1			SELM			STOM
ATP5L	NDUFA4			NNMT			IGFBP2
ATP5E	ADAMTS4			SEPT7			ACTA2
CCL11	SH3BGRL			WBP5			MYH9
PLAUR	MAP3K20			NFIB			POMP
ATP5I	ACTN4			LIMA1			EPAS1
NFATC2	TNS1			PLTP			CD59
SELK	PPP1R12A			UGDH			MYL12A
ERRFI1	CHCHD10			USP53			ARPC5
C14orf2	ZNF331			SEPP1			LGALS1
SLPI	CPM			ATP5J			ATP5F1B
TCEB2	KLF2			CTSK			C20orf27
VIMP	MTHFD2			DKK1			LAMA4
IGFBP6	CRISPLD2			PTRF			CCND1
PLIN2	CD151			RGCC			CD248
CTGF	CYCS			EMP3			TUBA1B
ZFP36L1	RGS16			SMIM14			VAMP5
CXCL1	MKNK2			PMP22			IFITM1
ARID5B	CKB			F10			COL5A2
GREM1	TOB1			SH3BGRL3			PRSS23
PLAC9	FHL1			CXCL12			ID1
RND3	HCFC1R1			PRSS23			ASPN
GAS6	MT1M			UGP2			
MEDAG	BTG2			HAS2			
ZFP36L2	C12orf75			RARRES1			
TNFAIP3	PDK4			C7			
MT2A	ATF3			PTN			
ACSL4	SERTAD1						
DKK1	ATP1B3						
RARRES1	HES1						
TIMP1	MT1X						
IGFBP5	CREM						
G0S2	MT1A						
CXCL8	IL6						
	FKBP5						

	MT1E						

Supplementary Table 4. List of oligonucleotide primers for qRT-PCR.

Genes	Forward (+)	Reverse (-)
SOX18	CTCGCTGGCCTGTACTACG	GCTGCAGTTGAGGTACTGGT
HOXB9	TACCTCACCAGGGACCGTAG	GGGAGGACTGGGGGTAATCT
NFIC	ATGTATTCGTCCCCGCTCTG	ATGCTCAGCACGAAGTCCTC
NFE2L3	GGCAGCATAGCAAACTGCTC	GCTGGCTTTACTGCCAGAGA
CREB3L1	CCCAGATGGCTGGGAAATCA	CTTGGCATGGCCTGTCGT
MMP11	TCATGATCGACTTCGCCAGG	CAGTGGGTAGCGAAAGGTGT
WNT5A	TCCTCTCGCCCATGGAATTA	TGCAGTTCCACCTTCGATGT
POSTN	TCCCCGTGACTGTCTATAAGC	ACCTTGGTGACCTCTTCTTG
ACTB	CTACCTCATGAAGATCCTGACC	CACAGCTTCTCTTTGATGTCAC
